# PPARγ activation improves the microenvironment of perivascular adipose tissue and attenuates aortic stiffening in obesity

**DOI:** 10.1186/s12929-021-00720-y

**Published:** 2021-03-29

**Authors:** Ju-Yi Chen, Yi-Pin Wu, Chih-Yi Li, Huei-Fen Jheng, Ling-Zhen Kao, Ching-Chun Yang, Sy-Ying Leu, I-Chia Lien, Wen-Tsan Weng, Haw-Chih Tai, Yu-Wei Chiou, Ming-Jer Tang, Pei-Jane Tsai, Yau-Sheng Tsai

**Affiliations:** 1 Division of Cardiology, Department of Internal Medicine, National Cheng Kung University Hospital, Tainan, Taiwan, ROC; 2 Institute of Clinical Medicine, National Cheng Kung University, Tainan, Taiwan, ROC; 3 Research and Development Division, National Laboratory Animal Center, National Applied Research Laboratories, Taipei, Taiwan, ROC; 4 Department of Physiology, National Cheng Kung University, Tainan, Taiwan, ROC; 5 International Center for Wound Repair and Regeneration, National Cheng Kung University, Tainan, Taiwan, ROC; 6 Department of Medical Laboratory Science and Biotechnology, National Cheng Kung University, Tainan, Taiwan, ROC; 7 Center of Clinical Medicine Research, National Cheng Kung University Hospital, Tainan, Taiwan, ROC

**Keywords:** Arterial stiffness, Perivascular adipose tissue, Peroxisome proliferator-activated receptor γ, Obesity

## Abstract

**Background:**

Obesity-related cardiovascular risk, end points, and mortality are strongly related to arterial stiffening. Current therapeutic approaches for arterial stiffening are not focused on direct targeting within the vessel. Perivascular adipose tissue (PVAT) surrounding the artery has been shown to modulate vascular function and inflammation. Peroxisome proliferator-activated receptor γ (PPARγ) activation significantly decreases arterial stiffness and inflammation in diabetic patients with coronary artery disease. Thus, we hypothesized that PPARγ activation alters the PVAT microenvironment, thereby creating a favorable environment for the attenuation of arterial stiffening in obesity.

**Methods:**

Obese *ob/ob* mice were used to investigate the effect of PPARγ activation on the attenuation of arterial stiffening. Various cell types, including macrophages, fibroblasts, adipocytes, and vascular smooth muscle cells, were used to test the inhibitory effect of pioglitazone, a PPARγ agonist, on the expression of elastolytic enzymes.

**Results:**

PPARγ activation by pioglitazone effectively attenuated arterial stiffening in *ob/ob* mice. This beneficial effect was not associated with the repartitioning of fat from or changes in the browning of the PVAT depot but was strongly related to improvement of the PVAT microenvironment, as evidenced by reduction in the expression of pro-inflammatory and pro-oxidative factors. Pioglitazone treatment attenuated obesity-induced elastin fiber fragmentation and elastolytic activity and ameliorated the obesity-induced upregulation of cathepsin S and metalloproteinase 12, predominantly in the PVAT. In vitro, pioglitazone downregulated *Ctss* and *Mmp12* in macrophages, fibroblasts, and adipocytes—cell types residing within the adventitia and PVAT. Ultimately, several PPARγ binding sites were found in *Ctss* and *Mmp12* in Raw 264.7 and 3T3-L1 cells, suggesting a direct regulatory mechanism by which PPARγ activation repressed the expression of *Ctss* and *Mmp-12* in macrophages and fibroblasts.

**Conclusions:**

PPARγ activation attenuated obesity-induced arterial stiffening and reduced the inflammatory and oxidative status of PVAT. The improvement of the PVAT microenvironment further contributed to the amelioration of elastin fiber fragmentation, elastolytic activity, and upregulated expression of *Ctss* and *Mmp12*. Our data highlight the PVAT microenvironment as an important target against arterial stiffening in obesity and provide a novel strategy for the potential clinical use of PPARγ agonists as a therapeutic against arterial stiffness through modulation of PVAT function.

**Supplementary Information:**

The online version contains supplementary material available at 10.1186/s12929-021-00720-y.

## Background

Obesity is linked to elevated cardiovascular risk and adverse cardiac outcomes and may lead to fatal consequences [1]. Many studies, including ours, have shown that obese populations, regardless of age, have increased arterial stiffening, particularly in the central arteries [2–5]. The stiffness of elastic arteries such as the aorta is an independent predictor of cardiovascular events and mortality [6]. Therefore, the contribution of arterial stiffening to obesity-related cardiovascular risk, end points, and mortality cannot be neglected.

In humans, pulse wave velocity (PWV), the gold standard for measuring arterial stiffness, increases with age [7]. Genetic factors and metabolic diseases, predisposing vascular calcification, and changes in the protein composition of the vessel wall unfavorably affect arterial stiffness. There is evidence to support the hypothesis that body weight loss, induced by hypocaloric diets, physical exercise, weight loss medications, bariatric surgery, and weight-neutral risk modifications such as the use of lipid lowering, antidiabetic, and antihypertensive drugs, attenuates arterial stiffness [7–9]. However, none of these therapeutic strategies directly targets microenvironment within the vessel wall, raising the possibility of a more complicated mechanism through which obesity causes arterial stiffening.

Perivascular adipose tissue (PVAT) surrounding the blood vessel directly adjoins the lamina adventitia and serves as a structural support for the vasculature [10]. PVAT undergoes morphological and functional changes in obesity, displaying a range of features, from those of energy-consuming brown adipose tissue (BAT) to those of energy-storing white adipose tissue (WAT) [11, 12]. PVAT actively communicates with the arterial wall to regulate vascular function and inflammation [13, 14]. Our previous study also showed an increased state of inflammation and oxidative stress in PVAT, leading to inflammatory cell infiltration, elastin fiber fragmentation, and stiffening of the aorta [3]. Thus, the PVAT microenvironment might be a critical determinant for arterial stiffening, particularly in established obesity.

Peroxisome proliferator-activated receptor γ (PPARγ) is a nuclear receptor regulating adipocyte development and glucose homeostasis. Although PPARγ is mainly expressed in adipose tissue, it is also detectable in the vasculature, including endothelial cells (ECs), vascular smooth muscle cells (VSMCs), and macrophages [15, 16]. Treatment with the PPARγ agonists rosiglitazone and pioglitazone has been shown to significantly decrease arterial stiffness and arterial inflammation in diabetic patients with coronary artery disease, as well as in obese glucose-tolerant patients [17, 18]. Pioglitazone improves aortic elasticity and decreases inflammation in patients with rheumatoid arthritis [19]. PPARγ agonists may therefore protect against arterial stiffening by normalizing metabolic disorders and/or vascular inflammation in patients with metabolic and other related vascular diseases.

PPARγ activation has been shown to attenuate adipose tissue inflammation and mobilize fat from intra-abdominal to subcutaneous depots [20], resulting in a beneficial outcome in the fat microenvironment. Thus, improvement of the PVAT microenvironment by PPARγ agonists may provide a novel therapeutic strategy against arterial stiffening associated with obesity. In this study, we hypothesized that PPARγ activation results in both repartition of fat from the PVAT depot and reduction of PVAT inflammation, ultimately changing the PVAT microenvironment, rendering it favorable for preserving the integrity of elastic fibers. To test this hypothesis, we employed obese *ob/ob* mice, which presented with high pro-inflammatory and pro-oxidative PVAT volumes as well as aortic stiffening in our previous study [3]. The PPARγ agonist pioglitazone was used to examine the influence of PPARγ on the PVAT microenvironment and the aortic stiffening associated with obesity.

## Methods

### Animals

Leptin-deficient (*ob/ob*) and control C57BL/6 mice were obtained from National Laboratory Animal Center (Tainan, Taiwan) fed regular chow. Pioglitazone was administered by oral gavage (20 mg/kg/day) or powder mixed in regular chow (40 mg/kg/day) on eight-week-old male mice. Animals were kept in a specific-pathogen-free barrier facility and handled in accordance with procedures approved by the Institutional Animal Care and Use Committees of National Cheng Kung University.

### PWV measurement in mice

Mice were anesthetized with avertin and placed on a temperature controlled electrocardiogram board. Doppler spectrograms of aortic flow at the aortic arch and abdominal aortic site 25 mm apart were acquired with a 20 MHz pulsed Doppler probe. The aortic PWV value was calculated via dividing the distance between two signals detected by the time difference between two pulse arrivals relative to the R-wave of the electrocardiogram.

### Blood pressure measurements

Blood pressure (BP) was measured in conscious mice by the tail-cuff BP measurement system (BP-2000; Visitech Systems). The mice were trained for 2 days. BPs were measured 20 times per day for 3 consecutive days. The results were calculated as the average from three trials of five to ten measurements each day for 3 consecutive days.

### Histology

Aortic tissue along with PVAT was fixed in 10% formalin and embedded in paraffin. Paraffin-section was cut in 5 μm thick, stained with hematoxylin and eosin for histological examination. Van Gieson’s stain (Accustain elastic stain kit; Sigma-Aldrich) was used for evaluation of elastic fiber network and picrosirius red stain (Abcam) was used for examination of collagen network.

### Cell culture

Raw 264.7, 3T3-L1, and A7r5 cells were obtained from Food Industry Research and Development Institute, Taiwan, and were grown in Dulbecco’s Modified Eagle’s Media (DMEM) containing 10% fetal bovine serum (FBS). 3T3-L1 cell differentiation was induced by adding the induction cocktail containing insulin (1.7 µM), dexamethasone (1 µM) and 1-methyl-3-isobutyl-xanthine (IBMX, 0.5 M) to the medium. After 72 h, cells were changed to the medium containing DMEM with 10% FBS and insulin (1.7 µM). Cells were treated with various concentrations of pioglitazone (Cayman) for 20 h, and the cell lysates were used for RNA analysis and ChIP assay.

### RNA analysis

Aortic tissue and PVAT were collected separately and stored in RNAlater (Ambion). RNA was extracted using REzol reagent (Protech Technology) and mRNA was analyzed by SYBR green-based real-time quantitative RT-PCR (Applied Biosystems). Cyclophilin A was used as the internal control for each assay. Sequences of the primers for RT-PCR assays are shown in Additional file 1: Table S1. Each mRNA sample acquired from PVAT represents one *ob/ob* mouse or a pool of RNA samples from 2–3 control lean mice due to limited availability.

### Immunoblot analysis

Twenty micrograms of total proteins were separated in SDS-PAGE, transferred to PVDF membrane, and probed with antibodies against collagen type I (234167; Merck-Calbiochem), tropoelastin (MAB2503; Millipore), fibulin-4 (36475; Epitomics), fibulin-5 (12188–1-AP; Protein Tech) and α-tubulin (T5168; Sigma-Aldrich). Immunoreactive protein was detected by a chemiluminescence detection system (GE Healthcare, Pittsburgh, PA).

### Multiplexed immunohistochemistry

Paraffin-section containing aortic tissue and PVAT were deparaffinized, dehydrated, and boiled for epitope retrieval using an antigen retrieval buffer at pH = 6.0 (Opal 4-color IHC Kit, PerkinElmer). Tissue sections were blocked (StartingBlock™ T20 Blocking Buffer, 37539; Thermo-Fisher Scientific) for 1 h and incubated with primary antibodies (cathepsin S: ab18822, Abcam; MMP-12: PA5-13181, Thermo-Fisher Scientific; F4/80: ab6640, Abcam; CISD1: 16,006–1-AP, Proteintech; and NF-κB p65: SC-372, Santa Cruz) overnight at 4℃. The sections were incubated with HRP-conjugated secondary antibodies for 1 h and administrated with 50 × diluted Opal working solution for 10 min at room temperature. The following primary antibodies were stained with a repeat procedure including antigen stripping and blocking steps. DAPI was used to stain cell nuclei via adding mounting media containing fluoroshield with DAPI. The images were visualized by confocal microscopy (C1-Si, Nikon).

### In situ zymography

Frozen sections containing aortic tissue and PVAT were cut into 8 μm. Elastolytic enzymes activity was detected by in situ zymography. Slide administrated with 4% paraformaldehyde for 30 min was used as a negative control while one with diluted porcine pancreatic elastase was referred as a positive control. All slides were incubated with DQ-elastin substrate (EnzChek elastase activity assay kit, E-12056, Invitrogen) at 37 ℃ for 6 h. The fluorescence signal from digested DQ-elastin was detected by a fluorescence microscope (IX71, Olympus).

### Elastolytic activity

Aortic tissue and PVAT were dissected separately, homogenized and incubated with 10 × diluted reaction buffer and DQ-elastin substrate (EnzChek elastase activity assay kit, E-12056, Invitrogen) at 37 ℃. The fluorescence signal was detected using a microplate fluorometer at 485/538 nm every 5 min to 30 min and once per hour until 8 h. The curve was made from fluorescence detected, normalized to protein concentration over time, and the area under the curve represents the elastolytic activity.

### Chromatin immunoprecipitation (ChIP) assay

The procedure for ChIP was described previously [15]. In brief, the interaction between protein and DNA was fixed by using 1% formaldehyde for 10 min. Cells were harvested and sonicated to fragment DNA (average size of 200–500 bp). PPARγ antibody (ab41928, Abcam) was used to immunoprecipitate the PPARγ protein and DNA complexes. Potential PPARγ binding sites were amplified by specific primers (Additional file 1: Table S2) after reversing cross-linking.

### Enzyme-linked immunosorbent assay (ELISA)

Plasma levels of cathepsin S, MMP-12, and MCP-1 in mice were measured using respective mouse ELISA kits (CSB-EL006204MO, CUSABIO; ab246540, Abcam; and MJE00B, R&D Systems).

### Statistical analysis

Values are presented as means ± SEM. Student’s t-test was used for comparing two groups, and one-way-ANOVA was used for analyzing three groups followed by post hoc Fisher’s least significant difference for comparisons. Statistically significance was set at *P* < 0.05.

## Results

### Reduced aortic PWV in obese mice treated with pioglitazone

**Fig. 1:**
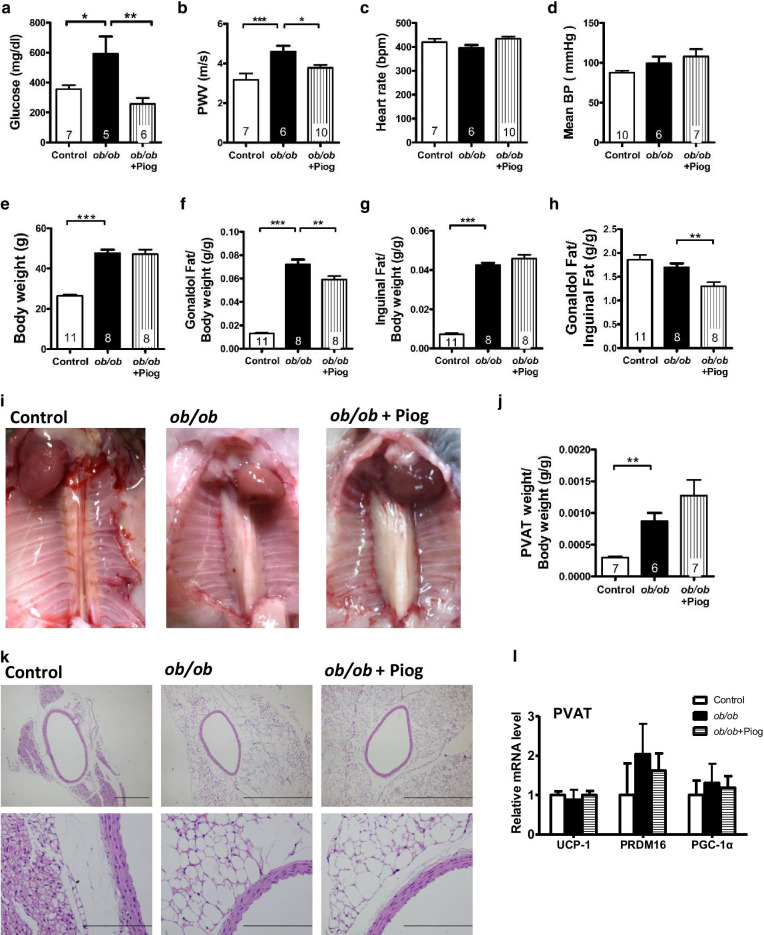
Biochemical analysis, fat deposits and PVAT of obese mice after pioglitazone treatment.** a** Plasma glucose, **b** aortic pulse wave velocity (PWV), **c** heart rate, **d** mean BP, **e** body weight, the ratio of **f** gonadal fat weight/body weight, **g** inguinal fat weight/body weight, and **h** gonadal/inguinal fat weight were measured in 3-month-old male control, *ob/ob*, and pioglitazone-treated *ob/ob* (*ob/ob* + Piog) mice. **i** PVAT was visualized in 3-month-old male mice and **j** its weight was normalized to body weight. Numbers inside the bar = *n* in each group. **k** Histological examination of aorta and PVAT. **l** Expression of brown adipocyte markers in the PVAT (*n* = 3, 5, and 6 for control, *ob/ob*, and *ob/ob* + Piog groups respectively). All data are resulted from 3-month-old male mice. *ob/ob* mice were treated with pioglitazone for 1 month starting at 2 months of age. *P < 0.05, **P < 0.01, and ***P < 0.001

Pioglitazone is widely used for the clinical treatment of diabetes, and we found that pioglitazone efficiently attenuated the increased plasma glucose level caused by obesity (Fig. 1a). However, pioglitazone did not change the increased plasma triglyceride and cholesterol levels (Additional file 1: Fig. S1a, b). Aortic PWV was significantly increased in *ob/ob* mice and reversed in the pioglitazone treatment group (Fig. 1b), suggesting that the aortas of *ob/ob* mice are stiffer than those of control lean mice and that pioglitazone reverses the aortic stiffness. Heart rate and BP, including mean, systolic, and diastolic BPs, did not differ between the three groups: lean controls, untreated *ob/ob* mice, and treated *ob/ob* mice. (Fig. 1c, d and Additional file 1: Fig. S1c, d). These results indicate that PPARγ activation attenuated obesity-induced aortic stiffening without influencing either BP or levels of circulating lipid.

### The effects of pioglitazone on fat deposits and PVAT in obese mice

The *ob/ob* mice have higher body weight and gonadal (intra-abdominal), and inguinal (subcutaneous) fat weights than lean control mice (Fig. 1e–g). While pioglitazone treatment did not change body weight, it significantly decreased gonadal fat weight and increased inguinal fat weight, resulting in a significant decrease in the gonadal/inguinal fat ratio (Fig. 1h). While PVAT is located within the peritoneum, pioglitazone treatment did not decrease PVAT mass when normalized to body weight or body length (Fig. 1i, j and Additional file 1: Fig. S1e). These results suggest a different mechanism is operating in the regulation of lipid homeostasis in the fat depots within the intra-abdominal cavity.

Histologically, the PVAT of lean control mice exhibited brown adipocyte-like features with multilocular lipid deposits, whereas the PVAT of *ob/ob* mice exhibited a white adipocyte-like appearance with markedly enlarged lipid droplets of unilocular lipid deposits (Fig. 1k). Although PPARγ agonists induce browning or beiging in subcutaneous WAT, pioglitazone treatment did not change the white adipocyte-like appearance in the PVAT of *ob/ob* mice (Fig. 1k). Expression of the brown adipocyte markers UCP1, PRDM16, and PGC-1α did not differ between the three groups (Fig. 1l), suggesting that the cellular type of adipocytes in PVAT may not be affected by pioglitazone treatment.

### Reduced inflammatory status in PVAT, but not in the aorta, after pioglitazone treatment

**Fig. 2:**
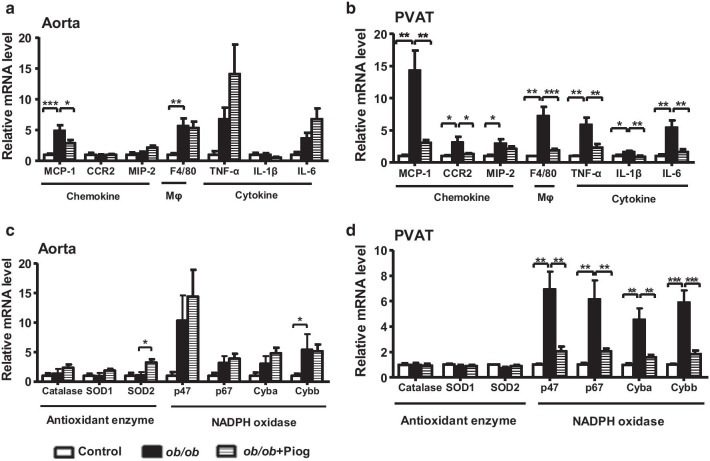
Expression of inflammatory mediators and reactive oxygen species-related enzymes in the aorta and PVAT. Expression of genes for chemokines, macrophage markers, and cytokines in the **a** aorta (*n* = 7, 5, and 6 for control, *ob/ob*, and *ob/ob* + Piog groups respectively) and **b** PVAT (*n* = 3, 5, and 6 for control, *ob/ob*, and *ob/ob* + Piog groups respectively). Expression of genes for ROS-scavenging enzymes and components of NADPH oxidase in the **c** aorta (*n* = 7, 5, and 6 for control, *ob/ob*, and *ob/ob* + Piog groups respectively) and **d** PVAT (*n* = 3, 5, and 6 for control, *ob/ob*, and *ob/ob* + Piog groups respectively). mRNA amount is expressed relative to the average expression in control mice. All data are resulted from 3-month-old male mice. *ob/ob* mice were treated with pioglitazone for 1 month starting at 2 months of age. *P < 0.05, **P < 0.01, and ***P < 0.001

The aorta of *ob/ob* mice showed significantly increased expression of monocyte chemotactic protein-1 (MCP-1) and macrophage marker (F4/80) and tended to have increased expression of tumor necrosis factor α (TNF-α) and interleukin 6 (IL-6) compared with those of lean control mice (Fig. 2a). However, the upregulation of these genes, except for MCP-1, were not reversed by pioglitazone treatment. In the PVAT, *ob/ob* mice exhibited significant upregulation of chemokines (MCP-1, CCR2, and MIP-2), a macrophage marker (F4/80), and cytokines (TNF-α, IL-1β, and IL-6) (Fig. 2b). Pioglitazone treatment effectively attenuated the upregulation of all of these genes. These results suggest that pioglitazone effectively attenuates PVAT inflammation but has less impact on aortic inflammation.

### Oxidative status declined in PVAT, but not in the aorta, after pioglitazone treatment

The expression of antioxidant enzymes, including catalase, superoxide dismutase I (SOD1), and superoxide dismutase II (SOD2), in the aorta was not significantly affected by obesity and pioglitazone treatment (Fig. 2c). By contrast, the expression of nicotinamide adenine dinucleotide phosphate (NADPH) oxidase components, including p47, p67, Cyba, and Cybb, in the aorta was increased by obesity, but this upregulation was not reversed by pioglitazone treatment. In the PVAT, the expression of the antioxidant enzymes catalase, SOD1, and SOD2 did not differ between the three groups (Fig. 2d). The expression of the NADPH oxidase components p47, p67, Cyba, and Cybb in the PVAT was significantly upregulated by obesity, and reversed after pioglitazone treatment. These results suggest that pioglitazone treatment effectively attenuates oxidative stress in the PVAT, but not in the aorta, of obese mice.

### Changes in elastic fiber and collagen homeostasis after pioglitazone treatment

**Fig. 3:**
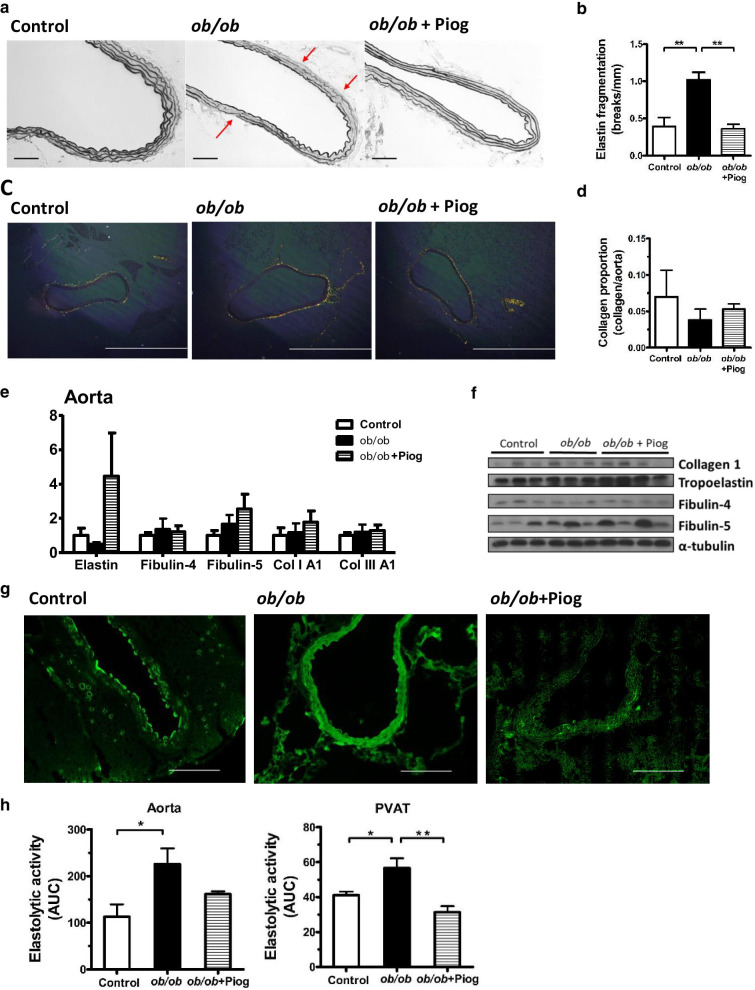
Elastin fiber fragmentation, collagen deposition, and elastolytic activity in the aorta. **a** Representative pictures for the elastic fiber network, and **b** quantification of elastin fiber breaks in the thoracic aortic media. Arrows indicate breaks in the elastin fiber. Each scale bar is 100 μm. **c** Collagen stain by the polarized view of picrosirius red-stained section and **d** quantification of collagen proportion of the aorta. Each scale bar is 1 mm. **e** Expression of collagen and elastic fiber components in the aorta (*n* = 7, 5, and 6 for control, *ob/ob*, and *ob/ob* + Piog groups respectively). mRNA amount is expressed relative to the average expression in control mice. **f** Immunoblot analysis on type I collagen, tropoelastin, fibulin-4/5 in the aorta. Each band represents a tissue extract from a single mouse. **g** In situ zymography for elastolytic activity in the frozen section. Each scale bar is 200 μm, **h** Tissue lysates of aorta and PVAT for elastolytic activity assay. The elastolytic activity was demonstrated as the area under the curve (AUC) of fluorescence with normalization to protein concentration over time. All data are resulted from 3-month-old male mice. *P < 0.05 and **P < 0.01

We further investigated the elasticity and stability of the vascular wall, and found that elastin fiber fragmentation was significantly increased in *ob/ob* mice, while pioglitazone treatment significantly reversed this effect (Fig. 3a, b). Collagen staining evinced by picrosirius red-stained sections under polarized light indicated that the collagen content did not differ between the three groups (Fig. 3c, d). The expression of elastin, elastic fiber components (fibulin-4 and fibulin-5), and collagen (type 1 and type 3 collagen) did not differ between the three groups (Fig. 3e, f). These results suggest that attenuation of aortic stiffness by pioglitazone in obesity can be attributed to the reversal of elastin fiber fragmentation.

### Decreased elastolytic activity after pioglitazone treatment

We directly tested the elastolytic activity in the aorta and PVAT. We first used in situ zymography by incubating frozen sections containing aorta and PVAT with the substrate DQ-elastin. We found a significant upregulation of the elastin digestion signal in both the aorta and PVAT of *ob/ob* mice compared with that in lean control mice (Fig. 3g). Pioglitazone treatment effectively attenuated the fluorescence from digested DQ-elastin in both the aorta and PVAT of *ob/ob* mice. We confirmed this finding by isolating tissue lysates from the aorta and PVAT for elastolytic activity assay. We found that the capability for digesting DQ-elastin was significantly higher in the aorta and PVAT of *ob/ob* mice (Fig. 3h) than those in control mice. While pioglitazone tended to decrease the elastolytic activity in the aorta, it significantly decreased the elastolytic activity in the PVAT of *ob/ob* mice.

### Downregulation of elastolytic enzymes in the aorta and PVAT after pioglitazone treatment

**Fig. 4:**
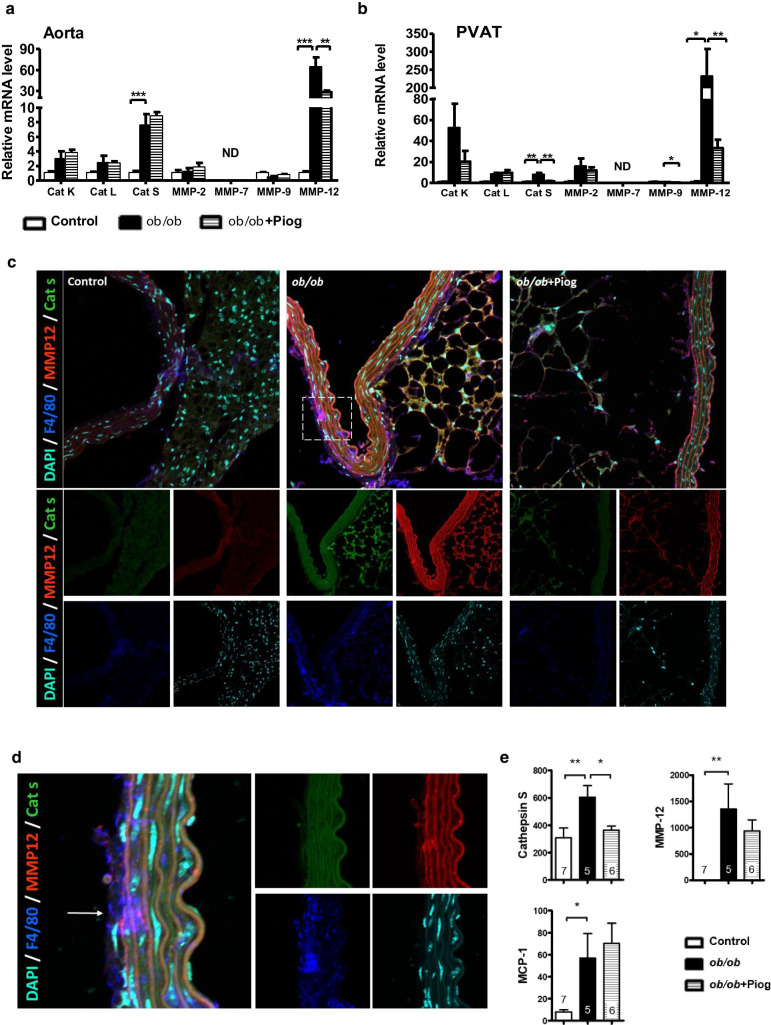
Expression and location of elastolytic enzymes in the aorta and PVAT. Expression of genes for cathepsins and MMPs in the **a** aorta (*n* = 7, 5, and 6 for control, *ob/ob*, and *ob/ob* + Piog groups respectively) and **b** PVAT (*n* = 3, 5, and 6 for control, *ob/ob*, *ob/ob* + Piog groups respectively). mRNA amount is expressed relative to the average expression in control mice. *P < 0.05, **P < 0.01, and ***P < 0.001. ND, not detectable. **c** Immunofluorescence staining for CTSS (green), MMP-12 (red) and F4/80 (blue) in the thoracic aorta and PVAT. The DAPI nuclear counterstain appears light blue. Magnification of the square in (**c**) is shown in (**d**). The while arrow indicates the location of elastic fiber break with increased signals of MMP-12 and F4/80. **e** Plasma levels of CTSS, MMP-12, and MCP-1 by ELISA (n = 7, 5, and 6 for control, *ob/ob*, and *ob/ob* + Piog groups respectively). All data are resulted from 3-month-old male mice. Original magnification × 400 for (**c**) and × 1200 for (**d**)

The imbalance between the activity of extracellular matrix (ECM) degradation enzymes, such as matrix metalloproteinases (MMPs) and cathepsins (CTSs), results in fragmentation of the elastic fibers in the aorta [3, 4]. We found that the expression of cathepsin K, cathepsin L, MMP-2, and MMP-9 did not differ in the aorta between the three groups (Fig. 4a). MMP-7 was not detectable in either aorta or PVAT. We found a significantly increased expression of both cathepsin S (CTSS) and MMP-12 in the aorta of *ob/ob* mice compared with that in lean control mice. While pioglitazone treatment attenuated the upregulation of MMP-12, it did not decrease the expression of CTSS. In the PVAT, the expression of cathepsin K, cathepsin L, MMP-2, and MMP-9 did not differ between groups (Fig. 4b). While *ob/ob* PVAT had significantly higher expression of CTSS and MMP-12, pioglitazone treatment effectively decreased this upregulation. These results indicate that pioglitazone decreases the expression of degradation enzymes for elastic fibers in the surrounding environment of aorta especially in the PVAT that may help to maintain the structure and integrity of the elastic fibers.

We used multiplexed immunofluorescence staining of the aorta and PVAT to examine the location and expression of CTSS, MMP-12, and the macrophage marker F4/80. Confocal microscopy showed that the expression of CTSS and MMP-12 was upregulated in both aorta and PVAT by obesity but was attenuated by pioglitazone (Fig. 4c and Additional file 1: Fig. S2). F4/80 was primarily upregulated in the aortic advantia and PVAT of *ob/ob* mice and was attenuated after pioglitazone treatment. We found that the location of elastic fiber breaks was associated with significant increases in F4/80 and MMP-12 (Fig. 4d). These results suggest a relationship between the upregulation of elastolytic enzymes and elastic fiber breakage. Moreover, plasma levels of CTSS, MMP-12, and MCP-1 were significantly increased in *ob/ob* mice (Fig. 4e). However, except for CTSS, pioglitazone did not significantly reduce the increased plasma levels of MMP-12 and MCP-1 in *ob/ob* mice. Thus, the effect of pioglitazone on attenuation of inflammatory mediators in circulation differs from that in local microenvironment.

### Inhibitory effect of pioglitazone on expression of CTSS and MMP-12

**Fig. 5:**
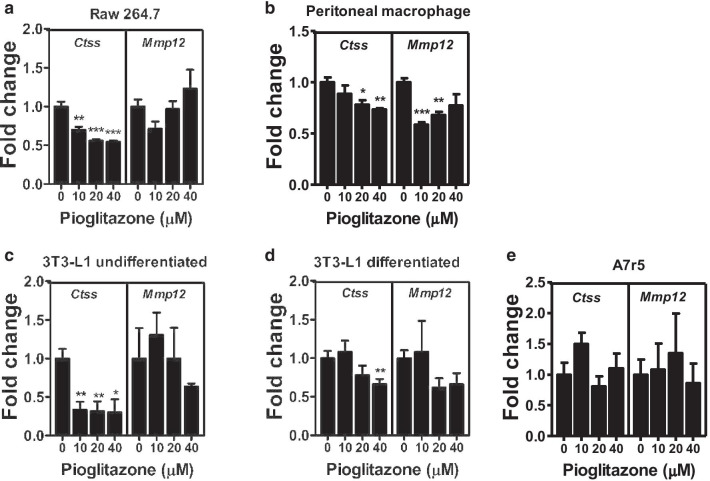
Effect of PPARγ activation on expression of *Ctss* and *Mmp12*.** a** Raw 264.7 cells, **b** peritoneal macrophages, **c** 3T3-L1 undifferentiated cells, **d** 3T3-L1 differentiated cells, and **e** A7r5 cells were treated with various concentrations of pioglitazone for 20 h. Expression of *Ctss* and *Mmp12* relative to the average expression of non-treatment control group. Data were collected from two independent experiments. *P < 0.05, **P < 0.01, and ***P < 0.001

**Fig. 6:**
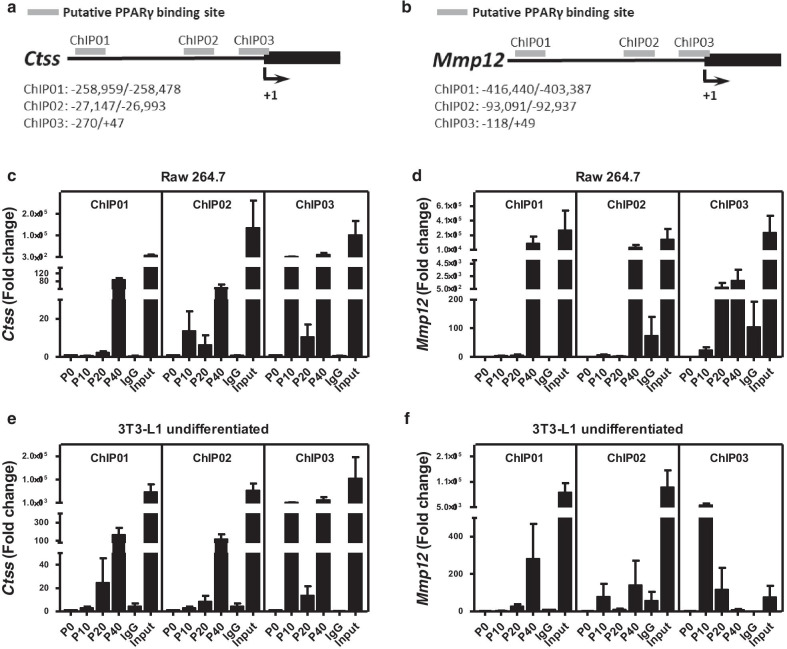
Binding of PPARγ in *Ctss* and *Mmp12*. ChIP assay in Raw 264.7 and 3T3-L1 undifferentiated cells treated with pioglitazone. **a**, **b** The schematic representation shows the location of primers flanking putative PPARγ-binding sites (grey rectangles) in *Ctss* and *Mmp12*. Sequences containing the potential PPARγ-binding sites in *Ctss* and *Mmp12* loci were amplified by real-time polymerase chain reaction in (**c**, **d**) Raw 264.7 and (**e**, **f**) 3T3-L1 undifferentiated cells. Data were collected from three independent experiments

To investigate which cell types within the aorta and PVAT might be responsible for the downregulation of CTSS and MMP-12, we treated various cell types, including macrophages (Raw 264.7 and peritoneal macrophages), fibroblasts (3T3-L1 cells), adipocytes (differentiated 3T3-L1 cells), and VSMCs (A7r5) with pioglitazone. While pioglitazone treatment induced expression of a reported PPARγ downstream gene, *CD36* or *aP2*, in all cell types tested (Additional file 1: Fig. S3), it downregulated CTSS in Raw 264.7, peritoneal macrophages, and undifferentiated and differentiated 3T3-L1 cells, but not in A7r5 cells (Fig. 5a–e). Furthermore, pioglitazone treatment only downregulated MMP-12 expression in peritoneal macrophages and had marginal effects on MMP-12 expression in other cell types (Fig. 5a–e). Searching previously reported ChIP–sequencing data [21], we found several potential PPARγ-binding sites in *Ctss* and *Mmp12* (Fig. 6a, b). ChIP-polymerase chain reaction (ChIP-PCR) assays in Raw 264.7 and 3T3-L1 cells showed that PPARγ bound to *Cd36*, *Ctss,* and *Mmp12* after pioglitazone treatment in both cell types (Fig. 6c–f and Additional file 1: Fig. S4), suggesting that *Ctss* and *Mmp12* are the direct targets of PPARγ in macrophages and fibroblasts.

### The role of mitoNEET in attenuation of obesity-mediated arterial stiffness by pioglitazone

**Fig. 7:**
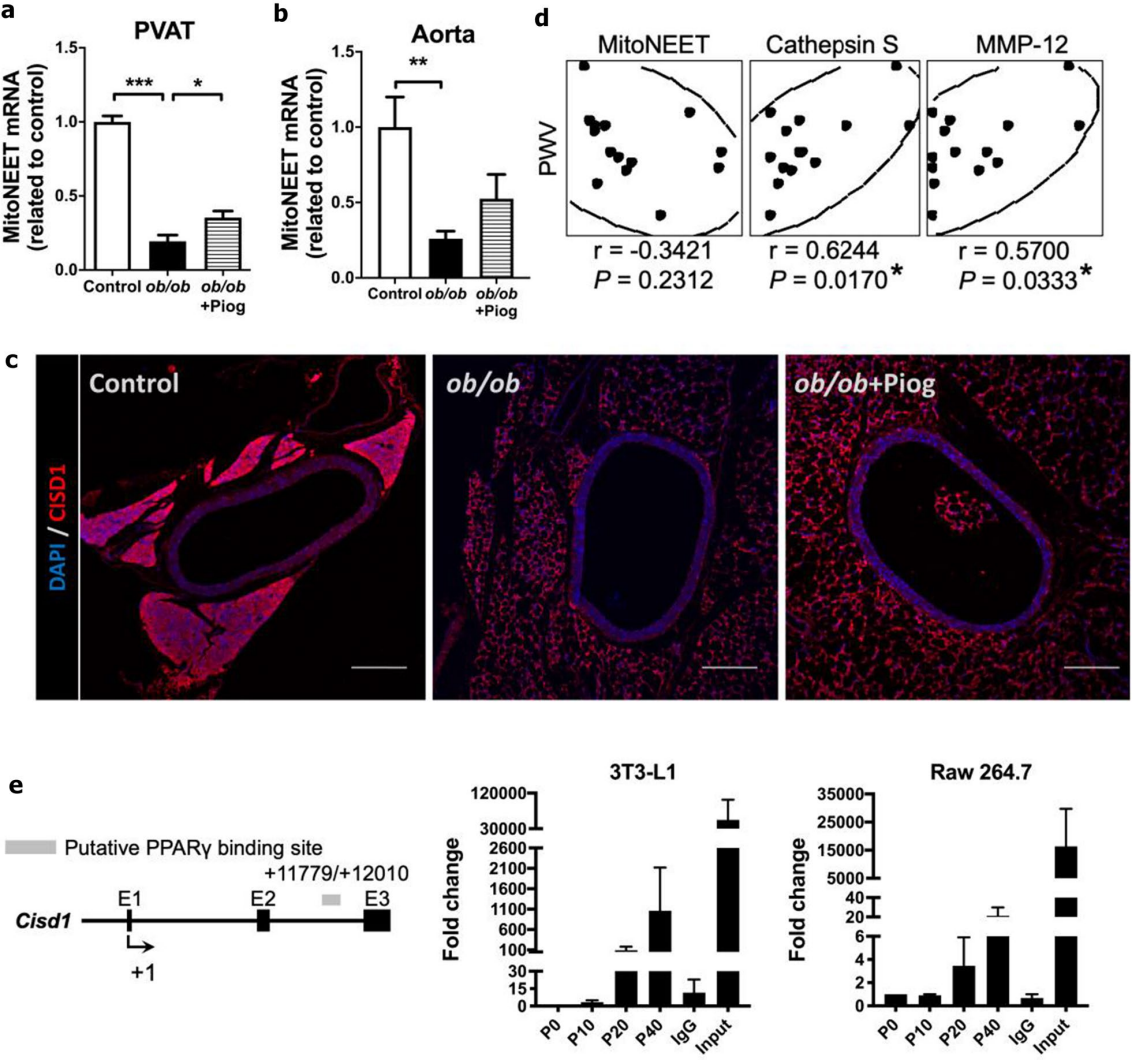
The role of mitoNEET in attenuation of obesity-mediated arterial stiffness by pioglitazone. Expression of *Cisd1* (coding for mitoNEET) in the **a** PVAT (n = 3, 5, and 6 for control, *ob/ob*, and *ob/ob* + Piog groups respectively) and **b** aorta (n = 7, 5, and 6 for control, *ob/ob*, and *ob/ob* + Piog groups respectively). *P < 0.05, **P < 0.01, and ***P < 0.001. **c** Immunofluorescence staining for mitoNEET (CISD1, red) and DAPI (blue) in the thoracic aorta and PVAT. Each scale bar is 200 μm. **d** The correlation between PWV and genes in the thoracic aorta (n = 5, 3, and 6 for control, *ob/ob*, and *ob/ob* + Piog groups respectively). Scatter plot illustrating the Spearman’s correlation of normalized reads per mouse between PWV and mitoNEET (*Cisd1*), CTSS (*Ctss*), and MMP-12 (*Mmp12*). Spearman’s rank correlation coefficients *r* and *P* value are provided in each plot. *P < 0.05. **e** ChIP assay in 3T3-L1 undifferentiated and Raw 264.7 cells treated with pioglitazone. The schematic representation shows the location of primers flanking putative PPARγ-binding sites (grey rectangles) in *Cisd1*. Sequence containing the potential PPARγ-binding sites in *Cisd1* loci was amplified by real-time polymerase chain reaction in 3T3-L1 undifferentiated and Raw 264.7 cells. Data were collected from three independent experiments

Because mitoNEET (also called CISD1), a mitochondrial outer membrane protein, has been implicated in the action of pioglitazone in PVAT [22], we conducted a series of experiments to examine the role of mitoNEET in attenuation of obesity-mediated arterial stiffness by pioglitazone. First, we examined mRNA and protein levels of mitoNEET in the aorta and PVAT. We found that mRNA levels of mitoNEET were significantly downregulated in the aorta and PVAT of *ob/ob* mice (Fig. 7a, b). Consistent with the study by Chang et al. [22], pioglitazone treatment significantly increased mitoNEET mRNA level in the PVAT of *ob/ob* mice, and tended to increase it in the aorta. Immunostaining consistently revealed that obesity downregulated mitoNEET in the PVAT, and pioglitazone treatment slightly reversed this downregulation (Fig. 7c). Second, we examined the correlation between aortic mitoNEET expression and PWV, but failed to observe the correlation between them (Fig. 7d). Interestingly, however, aortic expression of CTSS and MMP-12 significantly correlated with PWV. Third, we also demonstrated the binding of PPARγ to the genomic sequence of mitoNEET in the cell models. ChIP-PCR in both 3T3-L1 and Raw 264.7 cells showed that PPARγ bound to the genomic sequence of mitoNEET (Fig. 7e). These results suggest that mitoNEET can be one of the contributors in pioglitazone-mediated attenuation of arterial stiffness.

### The contribution of glucose and ECs in pioglitazone-mediated beneficial effect on improvement of arterial stiffness

**Fig. 8:**
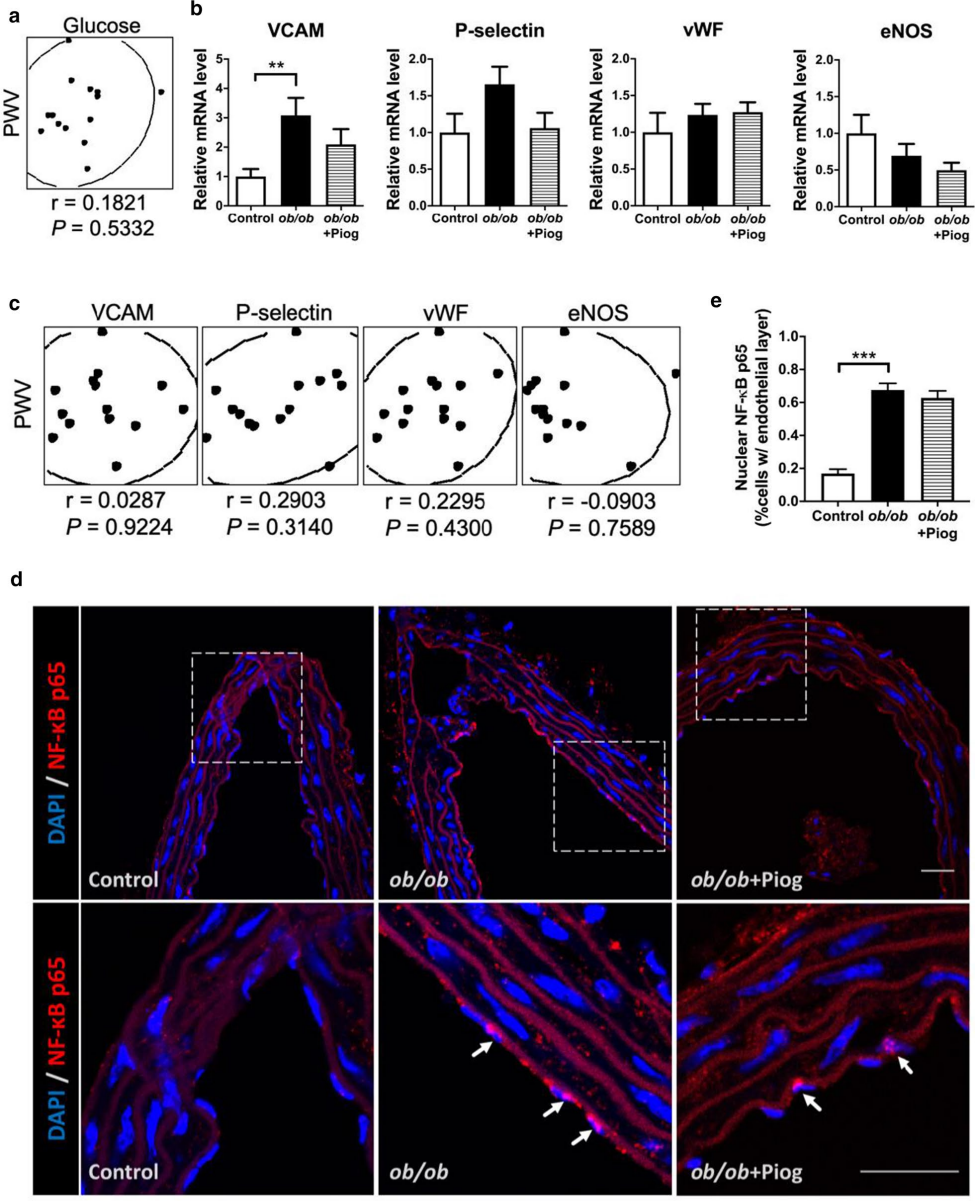
The contribution of glucose and ECs in pioglitazone-mediated beneficial effect on improvement of arterial stiffness.** a** The correlation between PWV and plasma glucose level in mice (n = 5, 3, and 6 for control, *ob/ob*, and *ob/ob* + Piog groups respectively). Scatter plot illustrating the Spearman’s correlation of normalized reads per mouse between PWV and plasma glucose level. **b** Expression of genes for endothelial markers in the aorta (n = 7, 5, and 6 for control, *ob/ob*, and *ob/ob* + Piog groups respectively). **P < 0.01. **c** The correlation between PWV and genes of endothelial markers in the thoracic aorta (n = 5, 3, and 6 for control, *ob/ob*, and *ob/ob* + Piog groups respectively). Scatter plot illustrating the Spearman’s correlation of normalized reads per mouse between PWV and VCAM (*Vcam*), P-selectin (*Selp*), vWF (*Vwf*), and eNOS (*Nos3*). Spearman’s rank correlation coefficients *r* and *P* value are provided in each plot. **d** Immunofluorescence staining for NF-κB p65 (red) and DAPI (blue) in the thoracic aorta. Each scale bar is 25 μm. Magnification of the square in upper panels is shown in lower panels. The while arrow indicates the location of positive nuclear staining for NF-κB. **e** Quantification of nuclear NF-κB p65 in the endothelial layer of thoracic aorta. ***P < 0.001

Because pioglitazone effectively reduced blood glucose levels in obese mice and glucose metabolism in ECs plays an important role in regulating vascular functional homeostasis, we then tested whether pioglitazone improves obesity-mediated arterial stiffness via the control of glucose metabolism and EC function. First, we examined the correlation between plasma glucose level and PWV, but did not observe the correlation between them (Fig. 8a). Second, we examined expression of several marker genes related to EC damage in aorta. Our results showed that obesity significantly upregulated VCAM, and tended to increase P-selectin and decrease eNOS (Fig. 8b). However, pioglitazone treatment did not significantly reverse these changes. Third, we performed the correlation analysis between aortic expression of these EC damage markers and PWV, but did not reveal the correlation of these genes with PWV (Fig. 8c). Fourth, we examined NF-κB nuclear localization in the EC. We found that obesity significantly increased NF-κB nuclear localization in the cells within endothelium, but pioglitazone did not reverse this increased NF-κB nuclear localization (Fig. 8d, e). Thus, while pioglitazone reduced blood glucose levels in obese mice, the contribution of glucose and EC function in pioglitazone-mediated beneficial effect on improvement of arterial stiffness may be modest.

## Discussion

In this study, we aimed to target the PVAT microenvironment as a novel therapeutic strategy against the arterial stiffening associated with obesity. We found that pioglitazone treatment reduced aortic PWV in obese mice. Although PPARγ activation remodeled the fat deposits and decreased the gonadal/inguinal ratio, it did not change the mass or the cellular type of adipocytes in PVAT. The attenuated inflammatory and oxidative status in the PVAT, but not in the aorta, contributed to the altered microenvironment surrounding the artery. PPARγ activation directly upregulated CTSS and MMP-12 expression in the cell types within the PVAT and aorta, accompanied by a reversal of elastin fiber fragmentation and a reduction of elastolytic activity in obesity. These data provide a new insight into the role of the PVAT microenvironment in arterial stiffening in obesity. The effect of PPARγ agonists on the modulation of PVAT function thus has the potential to be used as a therapeutic strategy against arterial stiffening.

Increased arterial stiffness, as reflected by enhanced PWV, is significantly associated with a high risk of cardiovascular mortality [6]. Recently, a joint scientific statement of the European Association indicated that the exclusion of obesity or fat distribution in current cardiovascular risk scores may underestimate the risk of early vascular aging in obesity [8]. In a large epidemiological study, obesity was found to be an independent predictor of cardiovascular risk, particularly in coronary heart disease and stroke [1]. A systematic search was conducted of human studies from the 1950s up to 2014 in many databases, and meta-analysis suggested that weight loss may reduce PWV [9]. The increased PWV is independent of age, sex, or BP in obese patients [23]. These data support the contention that obesity is an independent factor in arterial stiffness. Thus, these epidemiological observations suggest that obesity is an important factor in the development of arterial stiffening.

Hyperglycemia, tightly linked to vascular function, triggered endothelial dysfunction via reduction in nitric oxide synthesis and deterioration of antioxidant defense mechanisms [24, 25]. In our model, pioglitazone treatment concurrently ameliorated hyperglycemia and arterial stiffness in *ob/ob* mice. However, no correlation between plasma glucose level and PWV was found. Moreover, the genes coding for EC damage markers were not significantly reversed by pioglitazone, and not correlated with PWV either. Lastly, pioglitazone treatment did not ameliorate the increased nuclear NF-κB in the ECs of *ob/ob* mice. These results suggest that attenuation of hyperglycemia and EC damage may not play significant roles in the protective effect of pioglitazone on aortic stiffening in obesity.

As predicted by this hypothesis, the arterial stiffness and PWV were increased in non-hypertensive diet-induced obese mice. Our previous and current findings indicated that aortic PWV was significantly increased in *ob/ob* mice [3] independent of changes in BP and heart rate. In a diet-induced obese mouse model, adipokine dysregulation was associated with arterial stiffness but not the development of hypertension [26]. An increase in NADPH oxidases (NOX) 4 in the mitochondria of VSMCs and vasculature was associated with arterial stiffness in aged hypercholesterolemic mice [27]. Canugovi et al*.* found that young transgenic mice with high mitochondrial oxidative stress caused by increased NOX4 expression in mitochondria showed an increase in intrinsic VSMC stiffness, suggesting that the enhancement of ROS is critical for the pathogenesis of aortic stiffening in aging [28]. We also found significant, consistent increases in the expression of NADPH oxidase subunits in the PVAT, though the increases were relatively modest in the aorta. Both the lack of PVAT and hypertrophic PVAT caused by a high-fat diet enhanced arterial stiffness in aging mice [22], suggesting that PVAT dysfunction plays an important role in the pathogenesis of arterial stiffness.

Different fat pads have distinct impacts on cardiovascular disease development. For example, accumulation of subcutaneous adipose tissue is not associated with increased cardiovascular risk in human [29]. Central or abdominal obesity caused by visceral adiposity is more closely related to arterial stiffness than increased body mass index, regardless of age [30, 31]. Other studies have reported changes in visceral adiposity as an independent factor of arterial pulse pressure and arterial stiffness in non-hypertensive [32] and type 2 diabetic patients [33]. Visceral fat accumulation and waist circumference are positively correlated with arterial stiffness but not intima-media thickness in men with type 2 diabetes [34]. Even at normal weight, increased visceral adiposity remains associated with enhanced PWV in patients with type 2 diabetes [35]. Our results indicated that the reduction of PWV under pioglitazone treatment was accompanied by a decrease in the gonadal/inguinal fat ratio without change in body weight. Thus, intra-abdominal fat is a critical factor in the development of arterial stiffness. Because PVAT is located within the abdomen, it is one of the intra-abdominal fat depots. We initially speculated that PPARγ activation may decrease PVAT fat mass. However, our results showed that pioglitazone treatment did not affect PVAT fat mass, suggesting that PVAT has different characteristics in response to PPARγ-mediated fat remodeling. Thus, obesity-mediated arterial stiffening is not simply determined by the PVAT fat mass.

The PVAT of lean control mice exhibited multilocular lipid deposits, whereas the PVAT in *ob/ob* mice had a white adipocyte-like appearance, with markedly enlarged lipid droplets of unilocular lipid deposits. These results suggest that PVAT exhibits the phenotypes of both WAT and BAT [36]. Functionally, PVAT has brown adipocyte-like features as its lipid clearance characteristics and maintains intravascular temperature in response to cold stimulation [37]. However, we did not find any difference in browning genes within the PVAT between lean and *ob/ob* mice. Pioglitazone treatment did not change the white adipocyte-like appearance, or the expression of brown adipocyte markers, of PVAT in *ob/ob* mice. These results suggest that PPARγ activation in PVAT, and its concomitant decrease in arterial stiffness is unlikely to be related to the browning of PVAT.

PVAT has a paracrine action on the VSMCs and ECs that contribute to the regulation of vessel tone under physiological conditions [38]. Loss of PVAT results in impaired vascular homeostasis and endothelial dysfunction [37]. Accumulating evidence from human and animal studies indicates that obesity modulates the function of PVAT. PVAT loses its anti-contractile properties, which are attributed to decreased vasodilator adipokines, increased the release of vasoconstrictor factors and increased oxidative factors in obesity and metabolic syndrome [39, 40]. Our data showed a significant upregulation of MCP-1 and F4/80, as well as macrophage infiltration, in both the aorta and PVAT in obesity. However, the expression of other pro-inflammatory factors, such as chemokines and cytokines, and the pro-oxidative enzyme NADPH oxidase was significantly increased in the PVAT but not in the aorta in obesity. These results suggest that the altered PVAT microenvironment is tightly linked to the pathogenesis of arterial stiffening in a paracrine manner in obesity. Thus, modulation of the PVAT microenvironment through reduction of pro-inflammatory and pro-oxidative factors may provide a novel therapeutic strategy against arterial stiffness in obesity.

Pioglitazone, a PPARγ agonist, has been widely used in antidiabetic therapy and is well known to remodel fat deposition, such as by attenuating adipose tissue inflammation and oxidative stress, to yield a healthier adipose tissue microenvironment [41]. Recently, the effect of PPARγ agonists on the modulation of the PVAT microenvironment has been reported. Pioglitazone diminishes oxidative damage and pro-inflammatory markers in the PVAT of apolipoprotein E-deficient (*ApoE*^*−/−*^) mice under fructose overload [42]. Similarly, pioglitazone-induced upregulation of mitoNEET prevented PVAT inflammation and was negatively associated with arterial stiffness in diet-induced obese mice [22]. We found that pioglitazone treatment diminished arterial stiffness, a condition which is associated with attenuation of increased inflammation and oxidative stress, predominantly in the PVAT. Thus, the effect of pioglitazone on the amelioration of arterial stiffening may be attributed to its beneficial role in changing the PVAT microenvironment.

Although mitoNEET is implicated as one of the contributors in pioglitazone-mediated attenuation of arterial stiffness [22], there are several discrepancies between the study by Chang et al. and ours. For example, Chang et al. applied not only hyperphagic (high-fat diet) but also aging (diet feeding for more than 48 wks) mouse model, whereas ours applied relatively young (3-month-old) hyperphagic *ob/ob* mice. Because aging is a key determinant of arterial stiffness, combination of both factors may complicate the role of pioglitazone in attenuation of obesity-induced arterial stiffness. In addition, while brown adipocyte-specific overexpression of mitoNEET inhibited diet-induced arterial stiffness accompanied by downregulation of inflammatory genes [22], the action of pioglitazone in mediating this axis was not directly tested in their study. In summary, although the role of mitoNEET in pioglitazone-mediated attenuation of arterial stiffness cannot be neglected, the potential executers, such as CTSS and MMP-12, which are involved in the aortic structure homeostasis are directly regulated by PPARγ and significantly correlated with arterial stiffness.

The elasticity and stability of the vascular wall are closely related to the two structural proteins, elastin and collagen. Elastin contributes to the elasticity and resilience of the arteries, while collagen is responsible for tensile strength. Excessive collagen deposition or insufficient elastin fiber connection leads to aortic stiffening [1, 2]. However, we did not find any changes in the expression of structural proteins in the arterial wall of *ob/ob* mice. Pioglitazone treatment did not affect the expression of these structural proteins. Using in situ zymograph assays, we further found that pioglitazone tended to decrease elastolytic activity in the aorta and significantly decreased this activity in the PVAT of *ob/ob* mice. These results suggest that obesity-induced arterial stiffening is due to the increased remodeling of the aortic wall rather than alterations in the aortic structural proteins. We found that pioglitazone treatment attenuated the enhanced elastin fragmentation in *ob/ob* aortas, accompanied by the reversal of increased expression of CTSS and MMP-12, predominantly in the PVAT, and to a lesser extent in the aorta, of *ob/ob* mice. These findings suggest that the attenuation of proteolysis resulting from changes in the PVAT microenvironment may be involved in the inhibitory effect of pioglitazone on reduction of aortic stiffness.

To further investigate the cause of increased proteolysis, we used immunostaining and found that the expression of CTSS and MMP-12 was increased in both the aorta and the PVAT of *ob/ob* mice. CTSS is predominantly expressed in monocytes and many other non-skeletal tissues. The mature protein cleaves MHC class II molecules in the endolysosomal compartment, and the secreted form can remodel components of the ECM, such as elastin, collagen, and fibronectin [22]. CTSS expressed in human atheroma produces elastinolytic activity at the sites of vascular matrix remodeling [43]. CTSS also regulates ECM degradation and mediates fibroblast transdifferentiation, thereby preserving left ventricular function after myocardial infarction [44]. We found that elastic fiber breakage is co-localized with the signals of F4/80 and MMP-12, indicating the involvement of MMP-12 and macrophages in elastin fiber breakage. Increased activity of MMP-12, predominantly produced by macrophages [45], is associated with atherosclerosis [46]. MMP-12 secreted by arterial VSMCs has been shown to be an essential mediator of chronic arterial stiffening with age [47]. We speculated that obesity-induced arterial remodeling is predominantly due to the CTSS and MMP-12 secreted from the cells in the local microenvironment within or surrounding the artery.

To investigate the causal relationship between PPARγ activation and the downregulation of *Ctss* and *Mmp12*, we used several cell models, including macrophages (Raw 264.7 and peritoneal macrophages), fibroblasts (3T3-L1 cells), adipocytes (differentiated 3T3-L1 cells), and VSMCs (A7r5). While PPARγ activation did not affect the expression of *Ctss* and *Mmp12* in the VSMCs, it downregulated *Ctss* and *Mmp12* in macrophages, fibroblasts, and adipocytes at various concentrations. We further calculated the blood concentration of C_max_ after oral administration of pioglitazone at 20 mg/kg in mice. According to European Public Assessment Report (EPAR) for pioglitazone published by European Medicines Agency (EMA), the C_max_ in human serum is typically 900 ng/mL (equal to 2.52 μM) after a 30-mg dose within 1–3 h [48]. In rats, the C_max_ can reach 32 μM in plasma after oral administration of pioglitazone at 10 mg/kg BW [49]. Although we did not find the pharmacokinetics of pioglitazone reported in mice, the C_max_ in mice is about 2 ~ 5 fold higher than that in rats [50, 51]. Therefore, the C_max_ after oral administration of pioglitazone at 20 mg/kg BW in mice is expected to be around 100 μM, which is comparable to the dosage we used in vitro. These data suggest that the effect of PPARγ activation on the reduction of CTSS and MMP-12 within the aorta may be mediated through a coordinated downregulation in these cell types, such as macrophages and adventitial fibroblasts residing within the adventitia and PVAT. To further investigate the possibility of a direct interaction between PPARγ and *Ctss* and *Mmp12*, ChIP analysis was conducted using Raw 264.7 and 3T3-L1 cells. Although the PPARγ response element in *Mmp12* could not be analyzed using promoter analysis [52], we found several PPARγ-binding sites in *Ctss* and *Mmp12*. Thus, PPARγ could directly bind to *Ctss* and *Mmp12* upon pioglitazone treatment in both macrophages and fibroblasts. These data suggest that the reduction of CTSS and MMP-12 expression under PPARγ agonism could be regulated via transcriptional repression.

In our study, the beneficial effects of pioglitazone on improvements of PVAT microenvironments and aortic stiffening remain elusive. Particularly, the inside (glucose and EC function) and outside (mitoNEET in PVAT) contributions cannot be neglected. Because pioglitazone is well known to remodel fat deposition and modulate fat microenvironment, we focused on the fat depot surrounding aorta, the PVAT. Interestingly, however, pioglitazone attenuated obesity-induced aortic stiffening, which is not related to changes in PVAT mass and browning. Instead, pro-inflammatory and pro-oxidative mediators in the PVAT are significantly attenuated by pioglitazone. Furthermore, we applied several cell models and found that PPARγ directly attenuated CTSS and MMP-12, particularly in those cells residing within the PVAT. We thus proposed that pioglitazone improves PVAT microenvironments and aortic stiffening in obesity via multiple PPARγ target genes in the cells, such as macrophages and fibroblasts, residing within the PVAT.

Regarding the roles of macrophages and fibroblasts in governing pioglitazone-prevented aortic stiffening caused by obesity, we found that pioglitazone effectively suppressed *Ctss* and *Mmp12* in macrophages and fibroblasts. Moreover, we found the binding of PPARγ to the genomic sequences of *Ctss* and *Mmp12*, as well as *Cisd1*, in both cell lines. Therefore, we speculated that macrophages and fibroblasts residing within the PVAT surrounding the aorta can respond to pioglitazone in attenuation of *Ctss* and *Mmp12* expression, as well as upregulation of *Cisd1* expression. Among the genes we tested in the cell models, pioglitazone has more prominent effect on inhibition of *Ctss*. Consistently, we also found a significant reduction in the circulating CTSS level after treatment of pioglitazone in *ob/ob* mice. Thus, the reduction in circulating CTSS may be contributed by the action of pioglitazone in these two cell types. These results raised a possibility that these two cell types may contribute to attenuation of aortic proteolysis factors in both local aortic microenvironment and systemic circulation for improvement of obesity-induced aortic stiffening. However, dissecting the relative contribution of these two cell types in governing pioglitazone-prevented aortic stiffening caused by obesity would further require a macrophage or fibroblast-specific PPARγ knockout. Nevertheless, we believe that providing the information regarding how an unacknowledged function of pioglitazone in obesity-mediated aortic stiffening to the field, particularly for the translation purpose in clinics, is a timely issue.

## Conclusions

The present study focused on pioglitazone treatment against arterial stiffening in obesity. Although PPARγ activation did not significantly alter the mass or browning of PVAT, it significantly attenuated the inflammatory and oxidative status of PVAT. The improvement of PVAT and the local microenvironment surrounding the artery by pioglitazone in obesity further contributed to the attenuation of elastin fiber fragmentation, elastolytic activity, and expression of elastic fiber degradation enzymes. This effect is likely to be mediated through the direct regulation of *Ctss* and *Mmp12* by PPARγ in macrophages and fibroblasts. Our data highlight a novel therapeutic strategy against arterial stiffening, involving targeting the PVAT microenvironment in obesity. This study also suggests the possibility of repurposing PPARγ agonists as clinical therapeutics for arterial stiffening, acting by modulating PVAT function.

## Supplementary Information

**Additional file 1.** Additional figures and tables.

## Data Availability

All data generated or analysed during this study are included in this published article and its supplementary information files.

## Abbreviations

BAT: Brown adipose tissue
BP: Blood pressure
CTSS: cathepsin S
IL-6: Interleukin 6
MCP-1: Monocyte chemotactic protein-1
MMPs: Matrix metalloproteinases
PPARγ: Peroxisome proliferator-activated receptor γ
PVAT: Perivascular adipose tissue
PWV: Pulse wave velocity
SOD: Superoxide dismutase
TNF-α: Tumor necrosis factor α
VSMCs: Vascular smooth muscle cells
WAT: White adipose tissue
