# Supplementary material for: PPARγ activation improves the microenvironment of perivascular adipose tissue and attenuates aortic stiffening in obesity
Source: J Biomed Sci. 2021 Mar 29;28:22. doi: 10.1186/s12929-021-00720-y (PMC8008548; doi:10.1186/s12929-021-00720-y)
Supplement: Supplementary file 1 — Additional file 1. Additional figures and tables. [file 12929_2021_720_MOESM1_ESM.pdf]

ANNEX I

SUMMARY OF PRODUCT CHARACTERISTICS

1. NAME OF THE MEDICINAL PRODUCT

Actos 15 mg tablets
Actos 30 mg tablets
Actos 45 mg tablets

2. QUALITATIVE AND QUANTITATIVE COMPOSITION

<u>Actos 15 mg tablets</u>
Each tablet contains 15 mg of pioglitazone (as hydrochloride).

Excipient with known effect
Each tablet contains 92.87 mg of lactose monohydrate (see section 4.4).

<u>Actos 30 mg tablets</u>
Each tablet contains 30 mg of pioglitazone (as hydrochloride).

Excipient with known effect
Each tablet contains 76.34 mg of lactose monohydrate (see section 4.4).

<u>Actos 45 mg tablets</u>
Each tablet contains 45 mg of pioglitazone (as hydrochloride).

Excipient with known effect
Each tablet contains 114.51 mg of lactose monohydrate (see section 4.4).

For the full list of excipients, see section 6.1.

3. PHARMACEUTICAL FORM

Tablet

<u>Actos 15 mg tablets</u>
The tablets are white to off-white, round, convex and marked ‘15’ on one face and ‘ACTOS’ on the other face.

<u>Actos 30 mg tablets</u>
The tablets are white to off-white, round, flat and marked ‘30’ on one face and ‘ACTOS’ on the other face.

<u>Actos 45 mg tablets</u>
The tablets are white to off-white, round, flat and marked ‘45’ on one face and ‘ACTOS’ on the other face.

4. CLINICAL PARTICULARS

4.1 Therapeutic indications

Pioglitazone is indicated as second or third line treatment of type 2 diabetes mellitus as described below:

as **monotherapy**

- in adult patients (particularly overweight patients) inadequately controlled by diet and exercise for whom metformin is inappropriate because of contraindications or intolerance

as **dual oral therapy** in combination with

- metformin, in adult patients (particularly overweight patients) with insufficient glycaemic control despite maximal tolerated dose of monotherapy with metformin
- a sulphonylurea, only in adult patients who show intolerance to metformin or for whom metformin is contraindicated, with insufficient glycaemic control despite maximal tolerated dose of monotherapy with a sulphonylurea.

as **triple oral therapy** in combination with

- metformin and a sulphonylurea, in adult patients (particularly overweight patients) with insufficient glycaemic control despite dual oral therapy.

Pioglitazone is also indicated for combination with insulin in type 2 diabetes mellitus adult patients with insufficient glycaemic control on insulin for whom metformin is inappropriate because of contraindications or intolerance (see section 4.4).

After initiation of therapy with pioglitazone, patients should be reviewed after 3 to 6 months to assess adequacy of response to treatment (e.g. reduction in HbA_{1c}). In patients who fail to show an adequate response, pioglitazone should be discontinued. In light of potential risks with prolonged therapy, prescribers should confirm at subsequent routine reviews that the benefit of pioglitazone is maintained (see section 4.4).

4.2 Posology and method of administration

<u>Posology</u>

Pioglitazone treatment may be initiated at 15 mg or 30 mg once daily. The dose may be increased in increments up to 45 mg once daily.

In combination with insulin, the current insulin dose can be continued upon initiation of pioglitazone therapy. If patients report hypoglycaemia, the dose of insulin should be decreased.

<u>Special populations</u>

Elderly
No dose adjustment is necessary for elderly patients (see section 5.2). Physicians should start treatment with the lowest available dose and increase the dose gradually, particularly when pioglitazone is used in combination with insulin (see section 4.4 Fluid retention and cardiac failure).

Renal impairment
No dose adjustment is necessary in patients with impaired renal function (creatinine clearance > 4 mL/min) (see section 5.2). No information is available from dialysed patients therefore pioglitazone should not be used in such patients.

Hepatic impairment
Pioglitazone should not be used in patients with hepatic impairment (see sections 4.3 and 4.4).

Paediatric population
The safety and efficacy of Actos in children and adolescents under 18 years of age have not been established. No data are available.

Method of administration

Pioglitazone tablets are taken orally once daily with or without food. Tablets should be swallowed with a glass of water.

4.3 Contraindications

Pioglitazone is contraindicated in patients with:

- hypersensitivity to the active substance or to any of the excipients listed in section 6.1
- cardiac failure or history of cardiac failure (NYHA stages I to IV)
- hepatic impairment
- diabetic ketoacidosis
- current bladder cancer or a history of bladder cancer
- uninvestigated macroscopic haematuria

4.4 Special warnings and precautions for use

Fluid retention and cardiac failure

Pioglitazone can cause fluid retention, which may exacerbate or precipitate heart failure. When treating patients who have at least one risk factor for development of congestive heart failure (e.g. prior myocardial infarction or symptomatic coronary artery disease or the elderly), physicians should start with the lowest available dose and increase the dose gradually. Patients should be observed for signs and symptoms of heart failure, weight gain or oedema; particularly those with reduced cardiac reserve. There have been post-marketing cases of cardiac failure reported when pioglitazone was used in combination with insulin or in patients with a history of cardiac failure. Patients should be observed for signs and symptoms of heart failure, weight gain and oedema when pioglitazone is used in combination with insulin. Since insulin and pioglitazone are both associated with fluid retention, concomitant administration may increase the risk of oedema. Post marketing cases of peripheral oedema and cardiac failure have also been reported in patients with concomitant use of pioglitazone and nonsteroidal anti-inflammatory drugs, including selective COX-2 inhibitors. Pioglitazone should be discontinued if any deterioration in cardiac status occurs.

A cardiovascular outcome study of pioglitazone has been performed in patients under 75 years with type 2 diabetes mellitus and pre-existing major macrovascular disease. Pioglitazone or placebo was added to existing antidiabetic and cardiovascular therapy for up to 3.5 years. This study showed an increase in reports of heart failure; however this did not lead to an increase in mortality in this study.

Elderly

Combination use with insulin should be considered with caution in the elderly because of increased risk of serious heart failure.

In light of age- related risks (especially bladder cancer, fractures and heart failure), the balance of benefits and risks should be considered carefully both before and during treatment in the elderly.

Bladder cancer

Cases of bladder cancer were reported more frequently in a meta-analysis of controlled clinical trials with pioglitazone (19 cases from 12,506 patients, 0.15%) than in control groups (7 cases from 10,212 patients, 0.07%) HR=2.64 (95% CI 1.11-6.31, p=0.029). After excluding patients in whom exposure to study drug was less than one year at the time of diagnosis of bladder cancer, there were 7 cases (0.06%) on pioglitazone and 2 cases (0.02%) in control groups. Epidemiological studies have also suggested a small increased risk of bladder cancer in diabetic patients treated with pioglitazone, although not all studies identified a statistically significant increased risk.

Risk factors for bladder cancer should be assessed before initiating pioglitazone treatment (risks include age, smoking history, exposure to some occupational or chemotherapy agents e.g. cyclophosphamide or prior radiation treatment in the pelvic region). Any macroscopic haematuria should be investigated before starting pioglitazone therapy.

Patients should be advised to promptly seek the attention of their physician if macroscopic haematuria or other symptoms such as dysuria or urinary urgency develop during treatment.

Monitoring of liver function

There have been rare reports of hepatocellular dysfunction during post-marketing experience (see section 4.8). It is recommended, therefore, that patients treated with pioglitazone undergo periodic monitoring of liver enzymes. Liver enzymes should be checked prior to the initiation of therapy with pioglitazone in all patients. Therapy with pioglitazone should not be initiated in patients with increased baseline liver enzyme levels (ALT > 2.5 x upper limit of normal) or with any other evidence of liver disease.

Following initiation of therapy with pioglitazone, it is recommended that liver enzymes be monitored periodically based on clinical judgement. If ALT levels are increased to 3 x upper limit of normal during pioglitazone therapy, liver enzyme levels should be reassessed as soon as possible. If ALT levels remain > 3 x the upper limit of normal, therapy should be discontinued. If any patient develops symptoms suggesting hepatic dysfunction, which may include unexplained nausea, vomiting, abdominal pain, fatigue, anorexia and/or dark urine, liver enzymes should be checked. The decision whether to continue the patient on therapy with pioglitazone should be guided by clinical judgement pending laboratory evaluations. If jaundice is observed, the medicinal product should be discontinued.

Weight gain

In clinical trials with pioglitazone there was evidence of dose related weight gain, which may be due to fat accumulation and in some cases associated with fluid retention. In some cases weight increase may be a symptom of cardiac failure; therefore weight should be closely monitored. Part of the treatment of diabetes is dietary control. Patients should be advised to adhere strictly to a calorie-controlled diet.

Haematology

There was a small reduction in mean haemoglobin (4% relative reduction) and haematocrit (4.1% relative reduction) during therapy with pioglitazone, consistent with haemodilution. Similar changes were seen in metformin (haemoglobin 3-4% and haematocrit 3.6–4.1% relative reductions) and to a lesser extent sulphonylurea and insulin (haemoglobin 1–2% and haematocrit 1–3.2% relative reductions) treated patients in comparative controlled trials with pioglitazone.

Hypoglycaemia

As a consequence of increased insulin sensitivity, patients receiving pioglitazone in dual or triple oral therapy with a sulphonylurea or in dual therapy with insulin may be at risk for dose-related hypoglycaemia, and a reduction in the dose of the sulphonylurea or insulin may be necessary.

Eye disorders

Post-marketing reports of new-onset or worsening diabetic macular oedema with decreased visual acuity have been reported with thiazolidinediones, including pioglitazone. Many of these patients reported concurrent peripheral oedema. It is unclear whether or not there is a direct association between pioglitazone and macular oedema but prescribers should be alert to the possibility of macular oedema if patients report disturbances in visual acuity; an appropriate ophthalmological referral should be considered.

Others

An increased incidence in bone fractures in women was seen in a pooled analysis of adverse reactions of bone fracture from randomised, controlled, double blind clinical trials in over 8100 pioglitazone and 7400 comparator treated patients, on treatment for up to 3.5 years.

Fractures were observed in 2.6% of women taking pioglitazone compared to 1.7% of women treated with a comparator. No increase in fracture rates was observed in men treated with pioglitazone (1.3%) versus comparator (1.5%).

The fracture incidence calculated was 1.9 fractures per 100 patient years in women treated with pioglitazone and 1.1 fractures per 100 patient years in women treated with a comparator. The observed excess risk of fractures for women in this dataset on pioglitazone is therefore 0.8 fractures per 100 patient years of use.

In the 3.5 year cardiovascular risk PROactive study, 44/870 (5.1%; 1.0 fractures per 100 patient years) of pioglitazone-treated female patients experienced fractures compared to 23/905 (2.5%; 0.5 fractures per 100 patient years) of female patients treated with comparator. No increase in fracture rates was observed in men treated with pioglitazone (1.7%) versus comparator (2.1%).

Some epidemiological studies have suggested a similarly increased risk of fracture in both men and women.

The risk of fractures should be considered in the long term care of patients treated with pioglitazone (see section 4.8).

As a consequence of enhancing insulin action, pioglitazone treatment in patients with polycystic ovarian syndrome may result in resumption of ovulation. These patients may be at risk of pregnancy. Patients should be aware of the risk of pregnancy and if a patient wishes to become pregnant or if pregnancy occurs, the treatment should be discontinued (see section 4.6).

Pioglitazone should be used with caution during concomitant administration of cytochrome P450 2C8 inhibitors (e.g. gemfibrozil) or inducers (e.g. rifampicin). Glycaemic control should be monitored closely. Pioglitazone dose adjustment within the recommended posology or changes in diabetic treatment should be considered (see section 4.5).

Actos tablets contain lactose monohydrate and therefore should not be administered to patients with rare hereditary problems of galactose intolerance, total lactase deficiency or glucose-galactose malabsorption.

4.5 Interaction with other medicinal products and other forms of interaction

Interaction studies have shown that pioglitazone has no relevant effect on either the pharmacokinetics or pharmacodynamics of digoxin, warfarin, phenprocoumon and metformin. Co-administration of pioglitazone with sulphonylureas does not appear to affect the pharmacokinetics of the sulphonylurea. Studies in man suggest no induction of the main inducible cytochrome P450, 1A, 2C8/9 and 3A4. *In vitro* studies have shown no inhibition of any subtype of cytochrome P450. Interactions with substances metabolised by these enzymes, e.g. oral contraceptives, cyclosporin, calcium channel blockers, and HMGCoA reductase inhibitors are not to be expected.

Co-administration of pioglitazone with gemfibrozil (an inhibitor of cytochrome P450 2C8) is reported to result in a 3-fold increase in AUC of pioglitazone. Since there is a potential for an increase in dose-related adverse events, a decrease in the dose of pioglitazone may be needed when gemfibrozil is concomitantly administered. Close monitoring of glycaemic control should be considered (see section 4.4)**.** Co-administration of pioglitazone with rifampicin (an inducer of cytochrome P450 2C8)

is reported to result in a 54% decrease in AUC of pioglitazone. The pioglitazone dose may need to be increased when rifampicin is concomitantly administered. Close monitoring of glycaemic control should be considered (see section 4.4).

4.6 Fertility, pregnancy and lactation

Pregnancy

There are no adequate human data to determine the safety of pioglitazone during pregnancy. Foetal growth restriction was apparent in animal studies with pioglitazone. This was attributable to the action of pioglitazone in diminishing the maternal hyperinsulinaemia and increased insulin resistance that occurs during pregnancy thereby reducing the availability of metabolic substrates for foetal growth. The relevance of such a mechanism in humans is unclear and pioglitazone should not be used in pregnancy.

Breast-feeding

Pioglitazone has been shown to be present in the milk of lactating rats. It is not known whether pioglitazone is secreted in human milk. Therefore, pioglitazone should not be administered to breast-feeding women.

Fertility

In animal fertility studies there was no effect on copulation, impregnation or fertility index.

4.7 Effects on ability to drive and use machines

Actos has no or negligible influence on the ability to drive and use machines. However patients who experience visual disturbance should be cautious when driving or using machines.

4.8 Undesirable effects

Tabulated list of adverse reactions

Adverse reactions reported in excess (> 0.5%) of placebo and as more than an isolated case in patients receiving pioglitazone in double-blind studies are listed below as MedDRA preferred term by system organ class and absolute frequency. Frequencies are defined as: very common (≥ 1/10); common (≥ 1/100 to < 1/10); uncommon (≥ 1/1,000 to < 1/100); rare (≥ 1/10,000 to< 1/1,000); very rare (< 1/10,000); not known (cannot be estimated from the available data). Within each system organ class, adverse reactions are presented in order of decreasing incidence followed by decreasing seriousness.

**Adverse reaction**	**Frequency of adverse reactions of pioglitazone by treatment regimen**				
		**Combination**			
	**Mono-therapy**	**with metformin**	**with sulpho-nylurea**	**with metformin and sulpho-nylurea**	**with insulin**
**Infections and infestations**					
upper respiratory tract infection	common	common	common	common	common
bronchitis					common
sinusitis	uncommon	uncommon	uncommon	uncommon	uncommon

Adverse reaction	Frequency of adverse reactions of pioglitazone by treatment regimen				
		Combination			
	Mono-therapy	with metformin	with sulpho-nylurea	with metformin and sulpho-nylurea	with insulin
**Neoplasms benign, malignant and unspecified (including cysts and polyps)**					
bladder cancer	uncommon	uncommon	uncommon	uncommon	uncommon
**Blood and lymphatic system disorders**					
anaemia		common			
**Immune System Disorders**					
hypersensitivity and allergic reactions[1]	not known	not known	not known	not known	not known
**Metabolism and nutrition disorders**					
hypo-glycaemia			uncommon	very common	common
appetite increased			uncommon		
**Nervous system disorders**					
hypo-aesthesia	common	common	common	common	common
headache		common	uncommon		
dizziness			common		
insomnia	uncommon	uncommon	uncommon	uncommon	uncommon
**Eye disorders**					
visual disturbance[2]	common	common	uncommon		
macular oedema	not known	not known	not known	not known	not known
**Ear and labyrinth disorders**					
vertigo			uncommon		
**Cardiac disorders**					
heart failure[3]					common
**Respiratory, thoracic and mediastinal disorders**					
dyspnoea					common
**Gastrointestinal disorders**					
flatulence		uncommon	common		
**Skin and subcutaneous tissue disorders**					
sweating			uncommon		

Adverse reaction	Frequency of adverse reactions of pioglitazone by treatment regimen				
		Combination			
	Mono-therapy	with metformin	with sulpho-nylurea	with metformin and sulpho-nylurea	with insulin
**Musculoskeletal and connective tissue disorders**					
fracture bone[4]	common	common	common	common	common
arthralgia		common		common	common
back pain					common
**Renal and urinary disorders**					
haematuria		common			
glycosuria			uncommon		
proteinuria			uncommon		
**Reproductive system and breast disorders**					
erectile dysfunction		common			
**General disorders and administration site conditions**					
Oedema[5]					very common
fatigue			uncommon		
**Investigations**					
weight increased[6]	common	common	common	common	common
blood creatine phospho-kinase increased				common	
increased lactic dehydro-genase			uncommon		
alanine aminotransferase increased[7]	not known	not known	not known	not known	not known

<u>Description of selected adverse reactions</u>

[1] Postmarketing reports of hypersensitivity reactions in patients treated with pioglitazone have been reported. These reactions include anaphylaxis, angioedema, and urticaria.

[2] Visual disturbance has been reported mainly early in treatment and is related to changes in blood glucose due to temporary alteration in the turgidity and refractive index of the lens as seen with other hypoglycaemic treatments.

[3] In controlled clinical trials the incidence of reports of heart failure with pioglitazone treatment was the same as in placebo, metformin and sulphonylurea treatment groups, but was increased when used in combination therapy with insulin. In an outcome study of patients with pre-existing major macrovascular disease, the incidence of serious heart failure was 1.6% higher with pioglitazone than with placebo, when added to therapy that included insulin. However, this did not lead to an increase in mortality in this study. In this study in patients receiving pioglitazone and insulin, a higher

percentage of patients with heart failure was observed in patients aged ≥ 65 years compared with those less than 65 years (9.7% compared to 4.0%). In patients on insulin with no pioglitazone the incidence of heart failure was 8.2% in those ≥ 65 years compared to 4.0% in patients less than 65 years. Heart failure has been reported with marketing use of pioglitazone, and more frequently when pioglitazone was used in combination with insulin or in patients with a history of cardiac failure (see section 4.4).

[4] A pooled analysis was conducted of adverse reactions of bone fractures from randomised, comparator controlled, double blind clinical trials in over 8,100 patients in the pioglitazone-treated groups and 7,400 in the comparator-treated groups of up to 3.5 years duration. A higher rate of fractures was observed in women taking pioglitazone (2.6%) versus comparator (1.7%). No increase in fracture rates was observed in men treated with pioglitazone (1.3%) versus comparator (1.5%). In the 3.5 year PROactive study, 44/870 (5.1%) of pioglitazone-treated female patients experienced fractures compared to 23/905 (2.5%) of female patients treated with comparator. No increase in fracture rates was observed in men treated with pioglitazone (1.7%) versus comparator (2.1%). Post-marketing, bone fractures have been reported in both male and female patients (see section 4.4)

[5] Oedema was reported in 6–9% of patients treated with pioglitazone over one year in controlled clinical trials. The oedema rates for comparator groups (sulphonylurea, metformin) were 2–5%. The reports of oedema were generally mild to moderate and usually did not require discontinuation of treatment.

[6] In active comparator controlled trials mean weight increase with pioglitazone given as monotherapy was 2–3 kg over one year. This is similar to that seen in a sulphonylurea active comparator group. In combination trials pioglitazone added to metformin resulted in mean weight increase over one year of 1.5 kg and added to a sulphonylurea of 2.8 kg. In comparator groups addition of sulphonylurea to metformin resulted in a mean weight gain of 1.3 kg and addition of metformin to a sulphonylurea a mean weight loss of 1.0 kg.

[7] In clinical trials with pioglitazone the incidence of elevations of ALT greater than three times the upper limit of normal was equal to placebo but less than that seen in metformin or sulphonylurea comparator groups. Mean levels of liver enzymes decreased with treatment with pioglitazone. Rare cases of elevated liver enzymes and hepatocellular dysfunction have occurred in post-marketing experience. Although in very rare cases fatal outcome has been reported, causal relationship has not been established.

Reporting of suspected adverse reactions
Reporting suspected adverse reactions after authorisation of the medicinal product is important. It allows continued monitoring of the benefit/risk balance of the medicinal product. Healthcare professionals are asked to report any suspected adverse reactions via the national reporting system listed in Appendix V.

4.9 Overdose

In clinical studies, patients have taken pioglitazone at higher than the recommended highest dose of 45 mg daily. The maximum reported dose of 120 mg/day for four days, then 180 mg/day for seven days was not associated with any symptoms.

Hypoglycaemia may occur in combination with sulphonylureas or insulin. Symptomatic and general supportive measures should be taken in case of overdose.

5. PHARMACOLOGICAL PROPERTIES

5.1 Pharmacodynamic properties

Pharmacotherapeutic group: Drugs used in diabetes, blood glucose lowering drugs, excl. insulins; ATC code: A10BG03.

Pioglitazone effects may be mediated by a reduction of insulin resistance. Pioglitazone appears to act via activation of specific nuclear receptors (peroxisome proliferator activated receptor gamma) leading to increased insulin sensitivity of liver, fat and skeletal muscle cells in animals. Treatment with pioglitazone has been shown to reduce hepatic glucose output and to increase peripheral glucose disposal in the case of insulin resistance.

Fasting and postprandial glycaemic control is improved in patients with type 2 diabetes mellitus. The improved glycaemic control is associated with a reduction in both fasting and postprandial plasma insulin concentrations. A clinical trial of pioglitazone vs. gliclazide as monotherapy was extended to two years in order to assess time to treatment failure (defined as appearance of $HbA_{1c} \geq 8.0\%$ after the first six months of therapy). Kaplan-Meier analysis showed shorter time to treatment failure in patients treated with gliclazide, compared with pioglitazone. At two years, glycaemic control (defined as $HbA_{1c} < 8.0\%$) was sustained in 69% of patients treated with pioglitazone, compared with 50% of patients on gliclazide. In a two-year study of combination therapy comparing pioglitazone with gliclazide when added to metformin, glycaemic control measured as mean change from baseline in HbA_{1c} was similar between treatment groups after one year. The rate of deterioration of HbA_{1c} during the second year was less with pioglitazone than with gliclazide.

In a placebo controlled trial, patients with inadequate glycaemic control despite a three month insulin optimisation period were randomised to pioglitazone or placebo for 12 months. Patients receiving pioglitazone had a mean reduction in HbA_{1c} of 0.45% compared with those continuing on insulin alone, and a reduction of insulin dose in the pioglitazone treated group.

HOMA analysis shows that pioglitazone improves beta cell function as well as increasing insulin sensitivity. Two-year clinical studies have shown maintenance of this effect.

In one year clinical trials, pioglitazone consistently gave a statistically significant reduction in the albumin/creatinine ratio compared to baseline.

The effect of pioglitazone (45 mg monotherapy *vs.* placebo) was studied in a small 18-week trial in type 2 diabetics. Pioglitazone was associated with significant weight gain. Visceral fat was significantly decreased, while there was an increase in extra-abdominal fat mass. Similar changes in body fat distribution on pioglitazone have been accompanied by an improvement in insulin sensitivity. In most clinical trials, reduced total plasma triglycerides and free fatty acids, and increased HDL-cholesterol levels were observed as compared to placebo, with small, but not clinically significant increases in LDL-cholesterol levels.

In clinical trials of up to two years duration, pioglitazone reduced total plasma triglycerides and free fatty acids, and increased HDL cholesterol levels, compared with placebo, metformin or gliclazide. Pioglitazone did not cause statistically significant increases in LDL cholesterol levels compared with placebo, whilst reductions were observed with metformin and gliclazide. In a 20-week study, as well as reducing fasting triglycerides, pioglitazone reduced post prandial hypertriglyceridaemia through an effect on both absorbed and hepatically synthesised triglycerides. These effects were independent of pioglitazone’s effects on glycaemia and were statistically significantly different to glibenclamide.

In PROactive, a cardiovascular outcome study, 5,238 patients with type 2 diabetes mellitus and pre-existing major macrovascular disease were randomised to pioglitazone or placebo in addition to existing antidiabetic and cardiovascular therapy, for up to 3.5 years. The study population had an average age of 62 years; the average duration of diabetes was 9.5 years. Approximately one third of

patients were receiving insulin in combination with metformin and/or a sulphonylurea. To be eligible patients had to have had one or more of the following: myocardial infarction, stroke, percutaneous cardiac intervention or coronary artery bypass graft, acute coronary syndrome, coronary artery disease, or peripheral arterial obstructive disease. Almost half of the patients had a previous myocardial infarction and approximately 20% had had a stroke. Approximately half of the study population had at least two of the cardiovascular history entry criteria. Almost all subjects (95%) were receiving cardiovascular medicinal products (beta blockers, angiotensin-converting enzyme (ACE) inhibitors, angiotensin II antagonists, calcium channel blockers, nitrates, diuretics, acetylsalicylic acid, statins, fibrates).

Although the study failed regarding its primary endpoint, which was a composite of all-cause mortality, non-fatal myocardial infarction, stroke, acute coronary syndrome, major leg amputation, coronary revascularisation and leg revascularisation, the results suggest that there are no long-term cardiovascular concerns regarding use of pioglitazone. However, the incidences of oedema, weight gain and heart failure were increased. No increase in mortality from heart failure was observed.

Paediatric population

The European Medicines Agency has waived the obligation to submit the results of studies with Actos in all subsets of the paediatric population in type 2 diabetes mellitus. See section 4.2 for information on paediatric use.

5.2 Pharmacokinetic properties

Absorption

Following oral administration, pioglitazone is rapidly absorbed, and peak plasma concentrations of unchanged pioglitazone are usually achieved 2 hours after administration. Proportional increases of the plasma concentration were observed for doses from 2–60 mg. Steady state is achieved after 4–7 days of dosing. Repeated dosing does not result in accumulation of the compound or metabolites. Absorption is not influenced by food intake. Absolute bioavailability is greater than 80%.

Distribution

The estimated volume of distribution in humans is 0.25 L/kg.

Pioglitazone and all active metabolites are extensively bound to plasma protein (> 99%).

Biotransformation

Pioglitazone undergoes extensive hepatic metabolism by hydroxylation of aliphatic methylene groups. This is predominantly via cytochrome P450 2C8 although other isoforms may be involved to a lesser degree. Three of the six identified metabolites are active (M-II, M-III, and M-IV). When activity, concentrations and protein binding are taken into account, pioglitazone and metabolite M-III contribute equally to efficacy. On this basis M-IV contribution to efficacy is approximately three-fold that of pioglitazone, whilst the relative efficacy of M-II is minimal.

In vitro studies have shown no evidence that pioglitazone inhibits any subtype of cytochrome P450. There is no induction of the main inducible P450 isoenzymes 1A, 2C8/9, and 3A4 in man.

Interaction studies have shown that pioglitazone has no relevant effect on either the pharmacokinetics or pharmacodynamics of digoxin, warfarin, phenprocoumon and metformin. Concomitant administration of pioglitazone with gemfibrozil (an inhibitor of cytochrome P450 2C8) or with rifampicin (an inducer of cytochrome P450 2C8) is reported to increase or decrease, respectively, the plasma concentration of pioglitazone (see section 4.5).

Elimination

Following oral administration of radiolabelled pioglitazone to man, recovered label was mainly in faeces (55%) and a lesser amount in urine (45%). In animals, only a small amount of unchanged pioglitazone can be detected in either urine or faeces. The mean plasma elimination half-life of unchanged pioglitazone in man is 5 to 6 hours and for its total active metabolites 16 to 23 hours.

Elderly

Steady state pharmacokinetics are similar in patients age 65 and over and young subjects.

Patients with renal impairment

In patients with renal impairment, plasma concentrations of pioglitazone and its metabolites are lower than those seen in subjects with normal renal function, but oral clearance of parent substance is similar. Thus free (unbound) pioglitazone concentration is unchanged.

Patients with hepatic impairment

Total plasma concentration of pioglitazone is unchanged, but with an increased volume of distribution. Intrinsic clearance is therefore reduced, coupled with a higher unbound fraction of pioglitazone.

5.3 Preclinical safety data

In toxicology studies, plasma volume expansion with haemodilution, anaemia, and reversible eccentric cardiac hypertrophy was consistently apparent after repeated dosing of mice, rats, dogs, and monkeys. In addition, increased fatty deposition and infiltration were observed. These findings were observed across species at plasma concentrations ≤ 4 times the clinical exposure. Foetal growth restriction was apparent in animal studies with pioglitazone. This was attributable to the action of pioglitazone in diminishing the maternal hyperinsulinaemia and increased insulin resistance that occurs during pregnancy thereby reducing the availability of metabolic substrates for foetal growth.

Pioglitazone was devoid of genotoxic potential in a comprehensive battery of *in vivo* and *in vitro* genotoxicity assays. An increased incidence of hyperplasia (males and females) and tumours (males) of the urinary bladder epithelium was apparent in rats treated with pioglitazone for up to 2 years.

The formation and presence of urinary calculi with subsequent irritation and hyperplasia was postulated as the mechanistic basis for the observed tumourigenic response in the male rat. A 24-month mechanistic study in male rats demonstrated that administration of pioglitazone resulted in an increased incidence of hyperplastic changes in the bladder. Dietary acidification significantly decreased but did not abolish the incidence of tumours. The presence of microcrystals exacerbated the hyperplastic response but was not considered to be the primary cause of hyperplastic changes. The relevance to humans of the tumourigenic findings in the male rat cannot be excluded.

There was no tumorigenic response in mice of either sex. Hyperplasia of the urinary bladder was not seen in dogs or monkeys treated with pioglitazone for up to 12 months.

In an animal model of familial adenomatous polyposis (FAP), treatment with two other thiazolidinediones increased tumour multiplicity in the colon. The relevance of this finding is unknown.

Environmental Risk Assessment (ERA):
No environmental impact is anticipated from the clinical use of pioglitazone.

6. PHARMACEUTICAL PARTICULARS

6.1 List of excipients

Carmellose calcium
Hyprolose
Lactose monohydrate
Magnesium stearate

6.2 Incompatibilities

Not applicable.

6.3 Shelf life

3 years.

6.4 Special precautions for storage

This medicinal product does not require any special storage conditions.

6.5 Nature and contents of container

Aluminium/aluminium blisters, packs of 14, 28, 30, 50, 56, 84, 90, 98, 112 and 196 tablets.

Not all pack sizes may be marketed.

6.6 Special precautions for disposal

No special requirements for disposal.

7. MARKETING AUTHORISATION HOLDER

Takeda Pharma A/S
Delta Park 45
2665 Vallensbaek Strand
Denmark

8. MARKETING AUTHORISATION NUMBER(S)

EU/1/00/150/001
EU/1/00/150/002
EU/1/00/150/003
EU/1/00/150/004
EU/1/00/150/005
EU/1/00/150/006
EU/1/00/150/007
EU/1/00/150/008
EU/1/00/150/009
EU/1/00/150/010
EU/1/00/150/011
EU/1/00/150/012
EU/1/00/150/013
EU/1/00/150/014

EU/1/00/150/015
EU/1/00/150/016
EU/1/00/150/017
EU/1/00/150/018
EU/1/00/150/019
EU/1/00/150/020
EU/1/00/150/021
EU/1/00/150/022
EU/1/00/150/023
EU/1/00/150/024
EU/1/00/150/025
EU/1/00/150/026
EU/1/00/150/027
EU/1/00/150/028
EU/1/00/150/029
EU/1/00/150/030

9. DATE OF FIRST AUTHORISATION/RENEWAL OF THE AUTHORISATION

Date of first authorisation: 13/10/2000
Date of latest renewal: 31/08/2010

10. DATE OF REVISION OF THE TEXT

Detailed information on this medicinal product is available on the website of the European Medicines Agency http://www.ema.europa.eu

ANNEX II

A. MANUFACTURERS RESPONSIBLE FOR BATCH RELEASE

B. CONDITIONS OR RESTRICTIONS REGARDING SUPPLY AND USE

C. OTHER CONDITIONS AND REQUIREMENTS OF THE MARKETING AUTHORISATION

D. CONDITIONS OR RESTRICTIONS WITH REGARD TO THE SAFE AND EFFECTIVE USE OF THE MEDICINAL PRODUCT

A. MANUFACTURERS RESPONSIBLE FOR BATCH RELEASE

Name and address of the manufacturers responsible for batch release

Takeda Ireland Limited
Bray Business Park
Kilruddery
County Wicklow
Ireland

Lilly S.A.
Avda. de la Industria 30
28108 Alcobendas (Madrid)
Spain

The printed package leaflet of the medicinal product must state the name and address of the manufacturer responsible for the release of the concerned batch.

B. CONDITIONS OR RESTRICTIONS REGARDING SUPPLY AND USE

Medicinal product subject to medical prescription.

C. OTHER CONDITIONS AND REQUIREMENTS OF THE MARKETING AUTHORISATION

- **Periodic safety update reports**

The requirements for submission of PSURs for this medicinal product are set out in the list of Union reference dates (EURD list) provided for under Article 107c(7) of Directive 2001/83/EC and any subsequent updates published on the European medicines web-portal.

D. CONDITIONS OR RESTRICTIONS WITH REGARD TO THE SAFE AND EFFECTIVE USE OF THE MEDICINAL PRODUCT

- **Risk management plan (RMP)**

The marketing authorisation holder (MAH) shall perform the required pharmacovigilance activities and interventions detailed in the agreed RMP presented in Module 1.8.2 of the marketing authorisation and any agreed subsequent updates of the RMP.

An updated RMP should be submitted:
- At the request of the European Medicines Agency;
- Whenever the risk management system is modified, especially as the result of new information being received that may lead to a significant change to the benefit/risk profile or as the result of an important (pharmacovigilance or risk minimisation) milestone being reached.

ANNEX III

LABELLING AND PACKAGE LEAFLET

A. LABELLING

PARTICULARS TO APPEAR ON THE OUTER PACKAGING

CARTON

1. NAME OF THE MEDICINAL PRODUCT

Actos 15 mg tablets

pioglitazone

2. STATEMENT OF ACTIVE SUBSTANCE(S)

Each tablet contains 15 mg pioglitazone (as hydrochloride).

3. LIST OF EXCIPIENTS

Contains lactose monohydrate. See leaflet for further information.

4. PHARMACEUTICAL FORM AND CONTENTS

14 tablets
28 tablets
30 tablets
50 tablets
56 tablets
84 tablets
90 tablets
98 tablets
112 tablets
196 tablets

5. METHOD AND ROUTE(S) OF ADMINISTRATION

Read the package leaflet before use.

Oral use.

6. SPECIAL WARNING THAT THE MEDICINAL PRODUCT MUST BE STORED OUT OF THE SIGHT AND REACH OF CHILDREN

Keep out of the sight and reach of children.

7. OTHER SPECIAL WARNING(S), IF NECESSARY

8. EXPIRY DATE

EXP

9. SPECIAL STORAGE CONDITIONS

10. SPECIAL PRECAUTIONS FOR DISPOSAL OF UNUSED MEDICINAL PRODUCTS OR WASTE MATERIALS DERIVED FROM SUCH MEDICINAL PRODUCTS, IF APPROPRIATE

11. NAME AND ADDRESS OF THE MARKETING AUTHORISATION HOLDER

Takeda Pharma A/S
Delta Park 45
2665 Vallensbaek Strand
Denmark

12. MARKETING AUTHORISATION NUMBER(S)

EU/1/00/150/007 14 tablets
EU/1/00/150/001 28 tablets
EU/1/00/150/016 30 tablets
EU/1/00/150/002 50 tablets
EU/1/00/150/009 56 tablets
EU/1/00/150/017 84 tablets
EU/1/00/150/018 90 tablets
EU/1/00/150/003 98 tablets
EU/1/00/150/025 112 tablets
EU/1/00/150/026 196 tablets

13. BATCH NUMBER

Lot

14. GENERAL CLASSIFICATION FOR SUPPLY

15. INSTRUCTIONS ON USE

16. INFORMATION IN BRAILLE

Actos 15 mg

17. UNIQUE IDENTIFIER – 2D BARCODE

2D barcode carrying the unique identifier included.

18. UNIQUE IDENTIFIER - HUMAN READABLE DATA

PC
SN
NN

PARTICULARS TO APPEAR ON THE OUTER PACKAGING

CARTON

1. NAME OF THE MEDICINAL PRODUCT

Actos 30 mg tablets

pioglitazone

2. STATEMENT OF ACTIVE SUBSTANCE(S)

Each tablet contains 30 mg pioglitazone (as hydrochloride).

3. LIST OF EXCIPIENTS

Contains lactose monohydrate. See leaflet for further information.

4. PHARMACEUTICAL FORM AND CONTENTS

14 tablets
28 tablets
30 tablets
50 tablets
56 tablets
84 tablets
90 tablets
98 tablets
112 tablets
196 tablets

5. METHOD AND ROUTE(S) OF ADMINISTRATION

Read the package leaflet before use.

Oral use.

6. SPECIAL WARNING THAT THE MEDICINAL PRODUCT MUST BE STORED OUT OF THE SIGHT AND REACH OF CHILDREN

Keep out of the sight and reach of children.

7. OTHER SPECIAL WARNING(S), IF NECESSARY

8. EXPIRY DATE

EXP

9. SPECIAL STORAGE CONDITIONS

10. SPECIAL PRECAUTIONS FOR DISPOSAL OF UNUSED MEDICINAL PRODUCTS OR WASTE MATERIALS DERIVED FROM SUCH MEDICINAL PRODUCTS, IF APPROPRIATE

11. NAME AND ADDRESS OF THE MARKETING AUTHORISATION HOLDER

Takeda Pharma A/S
Delta Park 45
2665 Vallensbaek Strand
Denmark

12. MARKETING AUTHORISATION NUMBER(S)

EU/1/00/150/008 14 tablets
EU/1/00/150/004 28 tablets
EU/1/00/150/019 30 tablets
EU/1/00/150/005 50 tablets
EU/1/00/150/010 56 tablets
EU/1/00/150/020 84 tablets
EU/1/00/150/021 90 tablets
EU/1/00/150/006 98 tablets
EU/1/00/150/027 112 tablets
EU/1/00/150/028 196 tablets

13. BATCH NUMBER

Lot

14. GENERAL CLASSIFICATION FOR SUPPLY

15. INSTRUCTIONS ON USE

16. INFORMATION IN BRAILLE

Actos 30 mg

17. UNIQUE IDENTIFIER – 2D BARCODE

2D barcode carrying the unique identifier included.

18. UNIQUE IDENTIFIER - HUMAN READABLE DATA

PC
SN
NN

PARTICULARS TO APPEAR ON THE OUTER PACKAGING

CARTON

1. NAME OF THE MEDICINAL PRODUCT

Actos 45 mg tablets

pioglitazone

2. STATEMENT OF ACTIVE SUBSTANCE(S)

Each tablet contains 45 mg pioglitazone (as hydrochloride).

3. LIST OF EXCIPIENTS

Contains lactose monohydrate. See leaflet for further information.

4. PHARMACEUTICAL FORM AND CONTENTS

14 tablets
28 tablets
30 tablets
50 tablets
56 tablets
84 tablets
90 tablets
98 tablets
112 tablets
196 tablets

5. METHOD AND ROUTE(S) OF ADMINISTRATION

Read the package leaflet before use.

Oral use.

6. SPECIAL WARNING THAT THE MEDICINAL PRODUCT MUST BE STORED OUT OF THE SIGHT AND REACH OF CHILDREN

Keep out of the sight and reach of children.

7. OTHER SPECIAL WARNING(S), IF NECESSARY

8. EXPIRY DATE

EXP

9. SPECIAL STORAGE CONDITIONS

10. SPECIAL PRECAUTIONS FOR DISPOSAL OF UNUSED MEDICINAL PRODUCTS OR WASTE MATERIALS DERIVED FROM SUCH MEDICINAL PRODUCTS, IF APPROPRIATE

11. NAME AND ADDRESS OF THE MARKETING AUTHORISATION HOLDER

Takeda Pharma A/S
Delta Park 45
2665 Vallensbaek Strand
Denmark

12. MARKETING AUTHORISATION NUMBER(S)

EU/1/00/150/011 14 tablets
EU/1/00/150/012 28 tablets
EU/1/00/150/022 30 tablets
EU/1/00/150/013 50 tablets
EU/1/00/150/014 56 tablets
EU/1/00/150/023 84 tablets
EU/1/00/150/024 90 tablets
EU/1/00/150/015 98 tablets
EU/1/00/150/029 112 tablets
EU/1/00/150/030 196 tablets

13. BATCH NUMBER

Lot

14. GENERAL CLASSIFICATION FOR SUPPLY

15. INSTRUCTIONS ON USE

16. INFORMATION IN BRAILLE

Actos 45 mg

17. UNIQUE IDENTIFIER – 2D BARCODE

2D barcode carrying the unique identifier included.

18. UNIQUE IDENTIFIER - HUMAN READABLE DATA

PC
SN
NN

MINIMUM PARTICULARS TO APPEAR ON BLISTERS OR STRIPS

BLISTER

1. NAME OF THE MEDICINAL PRODUCT

Actos 15 mg tablets

pioglitazone

2. NAME OF THE MARKETING AUTHORISATION HOLDER

Takeda (logo)

3. EXPIRY DATE

EXP

4. BATCH NUMBER

Lot

5. OTHER

FOR CALENDARISED PACKS:

Mon.
Tue.
Wed.
Thu.
Fri.
Sat.
Sun.

MINIMUM PARTICULARS TO APPEAR ON BLISTERS OR STRIPS

BLISTER

1. NAME OF THE MEDICINAL PRODUCT

Actos 30 mg tablets

pioglitazone

2. NAME OF THE MARKETING AUTHORISATION HOLDER

Takeda (logo)

3. EXPIRY DATE

EXP

4. BATCH NUMBER

Lot

5. OTHER

FOR CALENDARISED PACKS:

Mon.
Tue.
Wed.
Thu.
Fri.
Sat.
Sun.

MINIMUM PARTICULARS TO APPEAR ON BLISTERS OR STRIPS

BLISTER

1. NAME OF THE MEDICINAL PRODUCT

Actos 45 mg tablets

pioglitazone

2. NAME OF THE MARKETING AUTHORISATION HOLDER

Takeda (logo)

3. EXPIRY DATE

EXP

4. BATCH NUMBER

Lot

5. OTHER

FOR CALENDARISED PACKS:

Mon.
Tue.
Wed.
Thu.
Fri.
Sat.
Sun.

B. PACKAGE LEAFLET

Package Leaflet: Information for the patient

Actos 15 mg tablets
Actos 30 mg tablets
Actos 45 mg tablets
pioglitazone

Read all of this leaflet carefully before you start taking this medicine because it contains important information for you.
- Keep this leaflet. You may need to read it again.
- If you have any further questions, ask your doctor or pharmacist.
- This medicine has been prescribed for you only. Do not pass it on to others. It may harm them, even if their signs of illness are the same as yours.
- If you get any side effects, talk to your doctor or pharmacist. This includes any possible side effects not listed in this leaflet. See section 4.

What is in this leaflet

1. What Actos is and what it is used for
2. What you need to know before you take Actos
3. How to take Actos
4. Possible side effects
5. How to store Actos
6. Contents of the pack and other information

1. What Actos is and what it is used for

Actos contains pioglitazone. It is an anti-diabetic medicine used to treat type 2 (non-insulin dependent) diabetes mellitus in adults, when metformin is not suitable or has failed to work adequately. This is the diabetes that usually develops in adulthood.

Actos helps control the level of sugar in your blood when you have type 2 diabetes by helping your body make better use of the insulin it produces. Your doctor will check whether Actos is working 3 to 6 months after you start taking it.

Actos may be used on its own in patients who are unable to take metformin, and where treatment with diet and exercise has failed to control blood sugar or may be added to other therapies (such as metformin, sulphonylurea or insulin) which have failed to provide sufficient control of blood sugar.

2. What you need to know before you take Actos

Do not take Actos
- if you are allergic to pioglitazone or any of the other ingredients of this medicine (listed in section 6).
- if you have heart failure or have had heart failure in the past.
- if you have liver disease.
- if you have had diabetic ketoacidosis (a complication of diabetes causing rapid weight loss, nausea or vomiting).
- if you have or have ever had bladder cancer.
- if you have blood in your urine that your doctor has not checked.

Warnings and precautions
Talk to your doctor or pharmacist before taking Actos (also see section 4)

- if you retain water (fluid retention) or have heart failure problems, in particular if you are over 75 years old. If you take anti-inflammatory medicines which can also cause fluid retention and swelling, you must also tell your doctor.
- if you have a special type of diabetic eye disease called macular oedema (swelling of the back of the eye).
- if you have cysts on your ovaries (polycystic ovary syndrome). There may be an increased possibility of becoming pregnant because you may ovulate again when you take Actos. If this applies to you, use appropriate contraception to avoid the possibility of an unplanned pregnancy.
- if you have a problem with your liver or heart. Before you start taking Actos you will have a blood sample taken to check your liver function. This check may be repeated at intervals. Some patients with long-standing type 2 diabetes mellitus and heart disease or previous stroke who were treated with Actos and insulin experienced the development of heart failure. Inform your doctor as soon as possible if you experience signs of heart failure such as unusual shortness of breath or rapid increase in weight or localised swelling (oedema).

If you take Actos with other medicines for diabetes, it is more likely that your blood sugar could fall below the normal level (hypoglycaemia).

You may also experience a reduction in blood count (anaemia).

Broken bones
A higher number of bone fractures was seen in patients, particularly women taking pioglitazone. Your doctor will take this into account when treating your diabetes.

Children and adolescents
Use in children and adolescents under 18 years is not recommended.

Other medicines and Actos
Tell your doctor or pharmacist if you are taking, have recently taken or might take any other medicines, including medicines obtained without a prescription.

You can usually continue to take other medicines whilst you are being treated with Actos. However, certain medicines are especially likely to affect the amount of sugar in your blood:
- gemfibrozil (used to lower cholesterol)
- rifampicin (used to treat tuberculosis and other infections)

Tell your doctor or pharmacist if you are taking any of these. Your blood sugar will be checked, and your dose of Actos may need to be changed.

Actos with food and drink
You may take your tablets with or without food. You should swallow the tablets with a glass of water.

Pregnancy and breast-feeding
Tell your doctor if
- you are, you think you might be or are planning to become pregnant.
- you are breast-feeding or if you are planning to breast-feed your baby.

Your doctor will advise you to discontinue this medicine.

Driving and using machines
This medicine will not affect your ability to drive or use machines but take care if you experience abnormal vision.

Actos contains lactose monohydrate
If you have been told by your doctor that you have an intolerance to some sugars, contact your doctor before taking Actos.

3. How to take Actos

Always take this medicine exactly as your doctor or pharmacist has told you. Check with your doctor or pharmacist if you are not sure.

The usual starting dose is one tablet of 15 mg or of 30 mg of pioglitazone to be taken once daily. Your doctor may increase the dose to a maximum of 45 mg once a day. Your doctor will tell you the dose to take.

If you have the impression that the effect of Actos is too weak, talk to your doctor.

When Actos is taken in combination with other medicines used to treat diabetes (such as insulin, chlorpropamide, glibenclamide, gliclazide, tolbutamide) your doctor will tell you whether you need to take a smaller dose of your medicines.

Your doctor will ask you to have blood tests periodically during treatment with Actos. This is to check that your liver is working normally.

If you are following a special diet for diabetes, you should continue with this while you are taking Actos.

Your weight should be checked at regular intervals; if your weight increases, inform your doctor.

If you take more Actos than you should
If you accidentally take too many tablets, or if someone else or a child takes your medicine, talk to a doctor or pharmacist immediately. Your blood sugar could fall below the normal level and can be increased by taking sugar. It is recommended that you carry some sugar lumps, sweets, biscuits or sugary fruit juice.

If you forget to take Actos
Take Actos daily as prescribed. However if you miss a dose, just carry on with the next dose as normal. Do not take a double dose to make up for a forgotten tablet.

If you stop taking Actos
Actos should be used every day to work properly. If you stop using Actos, your blood sugar may go up. Talk to your doctor before stopping this treatment.

If you have any further questions on the use of this medicine, ask your doctor or pharmacist.

4. Possible side effects

Like all medicines, this medicine can cause side effects, although not everybody gets them.

In particular, patients have experienced the following serious side effects:

Heart failure has been experienced commonly (may affect up to 1 in 10 people) in patients taking Actos in combination with insulin. Symptoms are unusual shortness of breath or rapid increase in weight or localised swelling (oedema). If you experience any of these, especially if you are over the age of 65, seek medical advice straight away.

Bladder cancer has been experienced uncommonly (may affect up to 1 in 100 people) in patients taking Actos. Signs and symptoms include blood in your urine, pain when urinating or a sudden need to urinate. If you experience any of these, talk to your doctor as soon as possible.

Localised swelling (oedema) has also been experienced very commonly (may affect more than 1 in 10 people) in patients taking Actos in combination with insulin. If you experience this side effect, talk to your doctor as soon as possible.

Broken bones have been reported commonly (may affect up to 1 in 10 people) in female patients taking Actos and have also been reported in male patients (frequency cannot be estimated from the available data) taking Actos. If you experience this side effect, talk to your doctor as soon as possible.

Blurred vision due to swelling (or fluid) at the back of the eye has also been reported in patients taking Actos (frequency cannot be estimated from the available data). If you experience this symptom for the first time, talk to your doctor as soon as possible. Also, if you already have blurred vision and the symptom gets worse, talk to your doctor as soon as possible.

Allergic reactions have been reported with frequency not known (cannot be estimated from the available data) in patients taking Actos. If you have a serious allergic reaction, including hives and swelling of the face, lips, tongue, or throat that may cause difficulty in breathing or swallowing stop taking this medicine and talk to your doctor as soon as possible.

The other side effects that have been experienced by some patients taking Actos are:

common (may affect up to 1 in 10 people)
- respiratory infection
- abnormal vision
- weight gain
- numbness

uncommon (may affect up to 1 in 100 people)
- inflammation of the sinuses (sinusitis)
- difficulty sleeping (insomnia)

not known (frequency cannot be estimated from the available data)
- increase in liver enzymes
- allergic reactions

The other side effects that have been experienced by some patients when Actos is taken with other antidiabetic medicines are:

very common (may affect more than 1 in 10 people)
- decreased blood sugar (hypoglycaemia)

common (may affect up to 1 in 10 people)
- headache
- dizziness
- joint pain
- impotence
- back pain
- shortness of breath
- small reduction in red blood cell count
- flatulence

uncommon (may affect up to 1 in 100 people)
- sugar in urine, proteins in urine
- increase in enzymes
- spinning sensation (vertigo)
- sweating
- tiredness
- increased appetite

Reporting of side effects
If you get any side effects, talk to your doctor or pharmacist. This includes any possible side effects not listed in this leaflet. You can also report side effects directly via the national reporting system listed in Appendix V. By reporting side effects you can help provide more information on the safety of this medicine.

5. How to store Actos

Keep this medicine out of the sight and reach of children.

Do not use this medicine after the expiry date which is stated on the carton and the blister pack after "EXP". The expiry date refers to the last day of that month.

This medicine does not require any special storage precautions.

Do not throw away any medicines via wastewater or household waste. Ask your pharmacist how to throw away medicines you no longer use. These measures will help protect the environment.

6. Contents of the pack and other information

What Actos contains
– The active substance in Actos is pioglitazone.
  Each Actos 15 mg tablet contains 15 mg of pioglitazone (as hydrochloride).
  Each Actos 30 mg tablet contains 30 mg of pioglitazone (as hydrochloride).
  Each Actos 45 mg tablet contains 45 mg of pioglitazone (as hydrochloride).
– The other ingredients are lactose monohydrate, hyprolose, carmellose calcium and magnesium stearate. See section 2 "Actos contains lactose monohydrate".

What Actos looks like and contents of the pack
– Actos 15 mg tablets are white to off-white, round, convex tablets marked '15' on one face and 'ACTOS' on the other face.
– Actos 30 mg tablets are white to off-white, round, flat tablets marked '30' on one face and 'ACTOS' on the other face.
– Actos 45 mg tablets are white to off-white, round, flat tablets marked '45' on one face and 'ACTOS' on the other face.

The tablets are supplied in blister packs of 14, 28, 30, 50, 56, 84, 90, 98, 112 or 196 tablets. Not all the pack sizes may be marketed.

Marketing authorisation holder
Takeda Pharma A/S
Delta Park 45
2665 Vallensbaek Strand
Denmark

Manufacturer
Takeda Ireland Limited, Bray Business Park, Kilruddery, County Wicklow, Ireland.
Lilly S.A, Avda. de la Industria 30, 28108 Alcobendas (Madrid), Spain

For any information about this medicine, please contact the local representative of the Marketing Authorisation Holder:

België/Belgique/Belgien
Takeda Belgium
Tél/Tel: +32 2 464 06 11
takeda-belgium@takeda.com

България
Такеда България
Тел.: + 359 2 958 27 36; + 359 2 958 15 29

Česká republika
ELI LILLY ČR, s.r.o.
Tel: +420 234 664 111

Danmark
Takeda Pharma A/S
Tlf: +45 46 77 11 11

Deutschland
Takeda GmbH
Tel: +49 (0) 800 825 3325
medinfoEMEA@takeda.com

Eesti
Takeda Pharma AS
Tel: +372 617 7669

Ελλάδα
ΦΑΡΜΑΣΕΡΒ-ΛΙΛΛΥ Α.Ε.Β.Ε.
Τηλ: +30 210 629 4600

España
Lilly S.A.
Tel: +34 (91) 663 50 00

France
Takeda France SAS
Tél: +33 1 40 67 33 00
medinfoEMEA@takeda.com

Hrvatska
Takeda Pharmaceuticals Croatia d.o.o
Tel: +385 1 377 88 96

Ireland
Takeda Products Ireland Limited
Tel: +353 (0) 1 6420021

Lietuva
Eli Lilly Holdings Limited atstovybė
Tel. +370 (5) 2649600

Luxembourg/Luxemburg
Takeda Belgium
Tél/Tel: +32 2 464 06 11
takeda-belgium@takeda.com

Magyarország
Lilly Hungária Kft.
Tel.: +36 1 328 5100

Malta
Takeda Italia S.p.A.
Tel: +39 06 502601

Nederland
Eli Lilly Nederland B.V.
Tel: +31 (0) 30 60 25 800

Norge
Takeda AS
Tlf: +47 6676 3030
infonorge@takeda.com

Österreich
Takeda Pharma Ges.m.b.H.
Tel: +43(0)800 20 80 50

Polska
Takeda Pharma sp. z o.o.
Tel.: +48 22 608 13 00

Portugal
Lilly Portugal - Produtos Farmacêuticos, Lda
Tel: +351 21 412 6600

România
Eli Lilly România S.R.L.
Tel: +40 21 4023000

Slovenija
Takeda GmbH, Podružnica Slovenija
Tel: + 386 (0) 59 082 480

Ísland
Vistor hf.
Sími: +354 535 7000
vistor@vistor.is

Italia
Takeda Italia S.p.A.
Tel: +39 06 502601

Κύπρος
Takeda Pharma A/S
Τηλ: +45 46 77 11 11

Latvija
Eli Lilly Holdings Limited pārstāvniecība Latvijā
Tel: +371 6 7 364 000

Slovenská republika
Takeda Pharmaceuticals Slovakia s.r.o.
Tel: +421 (2) 20 602 600

Suomi/Finland
Oy Eli Lilly Finland Ab
Puh/Tel: +358 (0)9 8545250

Sverige
Takeda Pharma AB
Tel: +46 8 731 28 00
infosweden@takeda.com

United Kingdom
Takeda UK Ltd
Tel: +44 (0)1628 537 900

This leaflet was last approved in

Other sources of information

Detailed information on this medicine is available on the European Medicines Agency web site: http://www.ema.europa.eu.
